# Characterization of the basal angiosperm *Aristolochia fimbriata*: a potential experimental system for genetic studies

**DOI:** 10.1186/1471-2229-13-13

**Published:** 2013-01-24

**Authors:** Barbara J Bliss, Stefan Wanke, Abdelali Barakat, Saravanaraj Ayyampalayam, Norman Wickett, P Kerr Wall, Yuannian Jiao, Lena Landherr, Paula E Ralph, Yi Hu, Christoph Neinhuis, Jim Leebens-Mack, Kathiravetpilla Arumuganathan, Sandra W Clifton, Siela N Maximova, Hong Ma, Claude W dePamphilis

**Affiliations:** 1 Department of Biology, Institute of Molecular Evolutionary Genetics, and the Huck Institutes of the Life Sciences, 201 Life Sciences Building, Pennsylvania State University, University Park, PA 16802, USA; 2 USDA ARS PBARC, 64 Nowelo St., Hilo, HI 96720, USA; 3 Technische Universität Dresden, Institut für Botanik, D-01062, Dresden, Germany; 4 100 Jordan Hall, Clemson University, Clemson, SC, 29634, USA; 5 Department of Plant Sciences, University of Georgia, Athens, GA, 30602, USA; 6 Chicago Botanic Garden, Glencoe, IL, 27709, USA; 7 BASF Plant Science, 26 Davis Drive, Research Triangle Park, NC, 27709, USA; 8 Benaroya Research Institute at Virginia Mason, Flow Cytometry and Imaging Core Laboratory, 1201 Ninth Avenue, Seattle, WA, 98101, USA; 9 The Genome Institute,Washington University School of Medicine, 4444 Forest Park Boulevard, St. Louis, MO, 63108, USA; 10 Stephenson Research and Technology Center, Advanced Center for Genome Technology, University of Oklahoma, 101 David L. Boren Blvd, Norman, OK, 73019, USA; 11 Department of Horticulture, 421 Life Sciences Building, Pennsylvania State University, University Park, PA, 16802, USA; 12 State Key Laboratory of Genetic Engineering and the Institute of Plant Biology, the Center for Evolutionary Biology, the School of Life Sciences, Fudan University, Shanghai, 200433, China; 13 Institutes of Biomedical Sciences, Fudan University, Shanghai, 200032, China

## Abstract

**Background:**

Previous studies in basal angiosperms have provided insight into the diversity within the angiosperm lineage and helped to polarize analyses of flowering plant evolution. However, there is still not an experimental system for genetic studies among basal angiosperms to facilitate comparative studies and functional investigation. It would be desirable to identify a basal angiosperm experimental system that possesses many of the features found in existing plant model systems (e.g., *Arabidopsis* and *Oryza*).

**Results:**

We have considered all basal angiosperm families for general characteristics important for experimental systems, including availability to the scientific community, growth habit, and membership in a large basal angiosperm group that displays a wide spectrum of phenotypic diversity. Most basal angiosperms are woody or aquatic, thus are not well-suited for large scale cultivation, and were excluded. We further investigated members of Aristolochiaceae for ease of culture, life cycle, genome size, and chromosome number. We demonstrated self-compatibility for *Aristolochia elegans* and *A. fimbriata*, and transformation with a GFP reporter construct for *Saruma henryi* and *A. fimbriata*. Furthermore, *A. fimbriata* was easily cultivated with a life cycle of just three months, could be regenerated in a tissue culture system, and had one of the smallest genomes among basal angiosperms. An extensive multi-tissue EST dataset was produced for *A. fimbriata* that includes over 3.8 million 454 sequence reads.

**Conclusions:**

*Aristolochia fimbriata* has numerous features that facilitate genetic studies and is suggested as a potential model system for use with a wide variety of technologies. Emerging genetic and genomic tools for *A. fimbriata* and closely related species can aid the investigation of floral biology, developmental genetics, biochemical pathways important in plant-insect interactions as well as human health, and various other features present in early angiosperms.

## Background

Our present understanding of genetics, genomics, development, evolution, and physiology of living organisms has benefited greatly from work done in model genetic systems. Model systems provide more favorable conditions for observing phenomena and testing hypotheses than other systems afford. Models support inductive reasoning, in which one builds on the understanding of living organisms in general, based on observations made in a specific model organism [1]. Model organisms also provide a focus for researchers to work on a common system, resulting in collaborative and complementary efforts that can yield rapid progress and development of further resources. Models are crucial for understanding basic biological processes.

Model organisms should have several key attributes. They should share a number of characteristics of the taxon or process they are chosen to represent [1,2] and must be accessible [2] so that a broad community of scientists can utilize and develop them. Models used for developmental and genetic studies must also offer rapid development, short generation time, be amenable to large scale cultivation, have small seed size for easy storage of many genotypes, and provide ample tissue for experimentation [1]. Models should also support forward and reverse genetics, as is required for hypothesis testing [3], and have a small genome size to facilitate molecular genetics and genomics work, including genome sequencing and assembly [4,5]. Finally, for studying the evolution of development, models should have both conserved and unique features in comparison to related species, so that comparative studies can elucidate the mechanisms of phenotypic evolution [6].

**Figure 1:**
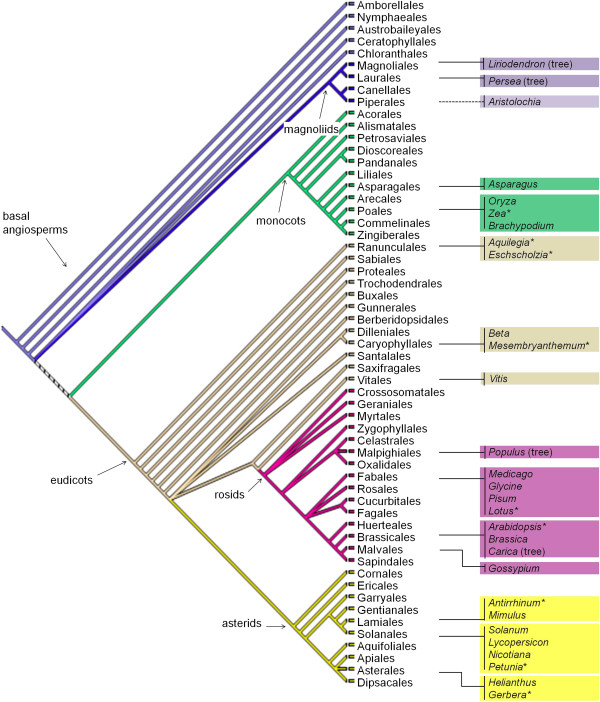
**Angiosperm phylogeny based on Stevens **[9]**, modified (http://www.mobot.org/MOBOT/research/APweb/welcome.html). **Important model systems and the proposed model, *Aristolochia fimbriata,* are shown next to the corresponding clade. *Liriodendron*, *Persea*, *Populus*, and *Carica *are tree species. Species that have been used as flower development models are indicated with an asterisk.

Our current understanding of angiosperm evolution has been shaped by multiple phylogenetic studies eg., [7-9] that provide the organismal context in which the evolution of any aspect of flowering plants is studied. Of particular interest to both basic and applied plant biology are changes leading to the success and diversification of angiosperm lineages, beginning with the early, mostly species-poor lineages of angiosperms (Table 1) previously known as the ANITA grade (Amborellaceae, Nymphaeales, Illiciaceae, Trimeniaceae, and Austrobaileyaceae), followed by the magnoliids (Figure 1). The genome of the shrub *Amborella trichopoda*, the likely singular living sister group to the rest of the living angiosperms, has been mapped [10] and sequenced (http://www.Amborella.org), providing an ideal root for comparative genomic studies of all other angiosperms [11]. The magnoliids contain four major branches and several thousand species (Table 1, Figure 1). Among the species in this lineage are the ornamental tree, *Liriodendron tulipfera*[12] and the fruit tree, *Persea americana*[13], as well as the economically important spice, black pepper (*Piper nigrum*) [14]. Taxa included in the magnoliids exhibit many features first appearing in basal angiosperms or angiosperms in general e.g., vessel elements, perianth bilateral symmetry, alkaloid chemistry, specialized pollination systems, and diverse forms of female gametophyte development [3,15,16]. Recent phylogenetic studies have suggested that the magnoliids are the sister clade and therewith the closest outgroup to the species-rich and highly diverged monocot and eudicot lineages [7-9].

**Table 1:** A summary of relevant basal angiosperm characteristics

**Order**	**Family**^**1,2**^	**# Genera / species**^**3**^	**Familiar genera**^**3**^	**Growth form**^**1,2**^	**Commercially available**
AMBORELLALES	Amborellaceae	1/1	*Amborella*	shrub	no
NYMPHAEALES	Nymphaeaceae	5/58	*Nymphaea*	aquatic herb	yes
			*Nuphar*		
			*Barclaya*		
			*Victoria*		
			*Euryale*		
	Cabombaceae	2/6	*Cabomba*	aquatic herb	yes
			*Brassenia*		
	Hydatellaceae	2/10	*Hydatella*	aquatic herb	no
			*Trithuria*		
AUSTROBAILEYALES	Austrobaileyaceae	1/2	*Austrobaileya*	liana	no
	Illiciaceae	3/92	*Illicium*	shrub, tree	yes
			*Schisandra*		
			*Kadsura*		
	Trimeniaceae	1-2/6	*Trimenia*	shrub, tree, liana	no
CERATOPHYLLALES	Ceratophyllaceae	1/6	*Ceratophyllum*	aquatic herb	yes
CHLORANTHALES	Chloranthaceae	4/75	*Chloranthus*	herb, shrub, tree	no
			*Ascarina*		
			*Hedyosmum*		
			*Sarcandra*		
MAGNOLIALES	Annonaceae	129/2220	*Annona*	shrub, tree, liana	yes
			*Guatteris*		
			*Xylopia*		
			*Uvaria*		
			*Polyalthia*		
			*Rollinia*		
			*Artabotrys*		
			*Asimina*		
	Eupomatiaceae	1/3	*Eupomatia*	shrub, tree	yes
	Magnoliaceae	2/227	*Magnolia*	shrub, tree	yes
			*Lioriodendron*		
	Degeneriaceae	1/2	*Degeneria*	tree	no
	Himantandraceae	1/2	*Galbulimima*	tree	no
	Myristicaceae	20/475	*Myristica*	shrub, tree	yes
			*Horsfieldia*		
			*Virola*		
			*Knema*		
LAURALES	Calycanthaceae	5/11	*Chimonanthus*	shrub, tree	yes
			*Calycanthus*		
			*Idiospermum*		
			*Sinocalycanthus*		
	Hernandiaceae	5/55	*Hernandia*	shrub, tree, liana	no
			*Illigera*		
	Lauraceae	50/200	*Laurus*	shrub, tree, parasitic vine	yes
			*Litsea*		
			*Ocotea*		
			*Cinnamomum*		
			*Cryptocarya*		
			*Persea*		
			*Lindera*		
			*Neolitsea*		
			*Nectandra*		
			*Phoebe*		
			*Apollonias*		
			*Beilschmiedia*		
			*Umbellularia*		
	Monimiaceae	22/200	*Doryphora*	shrub, liana	no
			*Peumus*		
			*Xymalos*		
			*Mollinedia*		
			*Tambourissa*		
			*Kibara*		
	Siparunaceae	2/75	*Siparuna*	shrub, tree	no
			*Glossocalyx*		
	Gomortegaceae	1/1	*Gomortega*	shrub, tree	no
	Atherosperma-taceae	6-7/16	*Atherosperma*	shrub, tree	no
			*Daphnandra*		
			*Doryphora*		
			*Dryadodaphne*		
			*Laurelia*		
			*Nemuaron*		
CANELLALES	Canellaceae	5/13	*Canella*	shrub, tree	yes
			*Cinnamodendron*		
			*Cinnamosma*		
	Winteraceae	4-7/60-90	*Drimys*	shrub, tree	yes
			*Zygogynum*		
			*Pseudowintera*		
			*Takhtajania*		
PIPERALES	Hydnoraceae	2/7	*Prosopanche*	parasitic herb	no
			*Hydnora*		
	Piperaceae	5/3600	*Peperomia*	herb	yes
			*Piper*	herb	yes
			*Zippelia*	herb	no
			*Manekia*	liana	no
			*Verhuellia*	herb	no
	Saururaceae	5/6	*Anemopsis*	herb	yes
			*Houttuynia*	herb	yes
			*Saururus*	herb	yes
			*Gymnotheca*	herb	no
	Lactoridaceae	1/1	*Lactoris*	shrub	no
	Aristolochiaceae	4/550	*Saruma (1)*	herb	yes
			*Asarum (ca. 86)*	herb	yes
			*Thottea (ca. 29)*	shrub	no
			*Aristolochia (ca. 450)*	herb, shrub, liana	yes

Familiar genera include representatives from the family and are not intended to comprise a comprehensive listing. Taxa are considered “commercially available” if one hundred plants or more can be purchased, inexpensively, and can be readily propagated from seed.

^1^[9].

^2^[17].

^3^Number of species shown for Aristolochiaceae genera from [18].

The rich diversity of basal angiosperms provides glimpses into early successful experiments in angiosperm adaptation [3,19-23]. Novel features which are otherwise constrained by function may have evolved more than once, through parallelism and convergence, such as the structural and developmental similarities of the inflorescence of the monocot *Acorus* with that found in some Piperales (Piperaceae, Saururaceae; members of Magnoliids) [24]. Basal lineages also retain evidence of “trials” of features that became genetically fixed in later lineages [25]. For example, perianth parts are quite variable in basal angiosperms, as well as in basal eudicots and basal monocots [26], and only became canalized later on.

Current flowering plant genetic models are all derived from the highly diverged monocot and eudicot lineages (Figure 1). Among them, the monocot models *Zea* and *Oryza* occur in Poaceae, which has specialized floral organs and an inflorescence found only in that family. Similarly, the current eudicot models are derived from the rosid (*Arabidopsis*) and asterid (e.g.*, Antirrhinum,* tomato) “core eudicot” lineages, each of which displays lineage-specific floral forms [25,27]. Studies in current plant models have led to the discovery of broadly homologous traits, including conservation of floral organ identity genes (ABC/quartet models) [28-30]. These homologies, and others observed in current plant models (Figure 1), have suggested hypotheses about the common ancestor of monocots and eudicots.

An understanding of the evolution of floral development or any fundamental process in flowering plants should include results from a basal angiosperm experimental model in which functional hypotheses can be tested [3,6,31-33]. Although the current model systems represent well the highly successful and derived lineages in which they occur, they do not represent the overall diversity of angiosperms. Information from basal lineages is necessary to better describe that diversity, to polarize the changes that occurred during angiosperm evolution, and to make functional inferences about the common ancestor of early angiosperms.

Current plant models have been selected to address particular questions, but very few are available for use both as genomic and as genetic models. Genomic resources, which emphasize plants with relatively small genomes, have been developed for tree wood and fruit species, including *Populus*[34], *Liriodendron*[12], *Persea*[35] and *Carica*[36]. However, woody species are too large at maturity and do not have short enough life cycles for general use in genetic experiments. Forward genetics requires a very small organism with a rapid life cycle, and is facilitated by the ability to self-pollinate individuals having desired characteristics. Reverse genetics requires manipulation of DNA or RNA in a targeted manner. Both benefit from a small genome, and transformability is essential for testing hypotheses about gene function. Therefore, we sought to identify a basal angiosperm species having as many important features of a model system as possible to support its potential development into an experimental system in genetics and genomics. We present these essential features in *Aristolochia fimbriata* - small size at maturity, rapid life cycling, self-compatibilty, small genome size, and transformability - along with relevant findings for other taxa evaluated in our study.

## Results

### Evaluation of potential models in basal angiosperm orders and families

**Figure 2:**
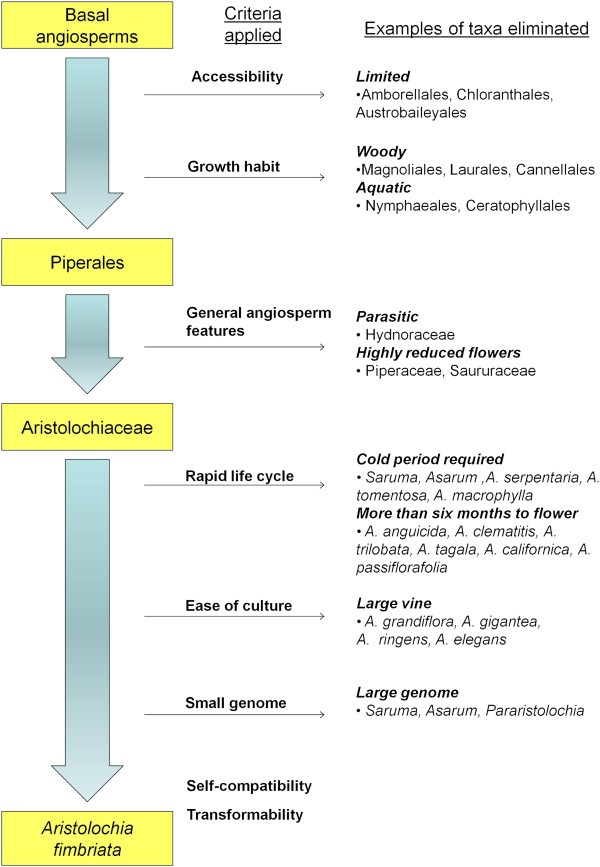
**Overall approach for selecting a basal angiosperm model system. **General criteria are indicated; for full description refer to methods. Taxa eliminated after initial application of each criterion are indicated. Some taxa may have been eliminated for more than one reason. For example, *Illicium*, in family Illiciaceae, is increasingly available in cultivation, unlike most Austrobaileyales, but was eliminated due to its woody growth habit. Many taxa in Piperales were generally accessible and of amenable growth habit, yet family Lactoridaceae was eliminated due to inaccessibility and woodiness. In genus *Aristolochia*, subgenus *Pararistolochia *was eliminated due to large genome size. Basal angiosperm family characteristics and those of Aristolochiaceae species cultivated are described in Tables 1 and 2.

We followed a formal selection process to identify a suitable candidate for an experimental organism among basal angiosperms (Figure 2). Many basal angiosperms are uncommon, with limited distribution, and often occur in families with only one genus and few species (Table 1). Taxa were considered readily accessible to a broad scientific community if they could be obtained commercially at a low price and could be readily propagated by seed. Those of limited availability were eliminated, including Amborellales (Amborellaceae), Austrobaileyales (Illiciaceae, Austrobaileyaceae, Trimeniaceae), and Chloranthales (Chloranthaceae) (Figure 2).

Next, we eliminated groups of plants sharing a growth habit associated with barriers for use as a genetic model. Woody plants requiring a year or more to attain maturity, as well as extensive space for cultivation, were eliminated (Figure 2). These included most Magnoliales (Myristicaceae, Magnoliaceae, Annonaceae, Himantandraceae, Degeneriaceae, Eupomatiaceae), Laurales (Lauraceae, Hernandiaceae, Monimiaceae, Atherospermataceae, Gomortegaceae, Siparunaceae, Calycanthaceae), and Cannellales (Cannellaceae, Winteraceae). The forest tree species, *Liriodendron* (Magnoliaceae) and the fruit tree *Persea* (Lauraceae), were among those eliminated due to woody habit and long generation time. Many of the families listed above would also be eliminated due to limited commercial availability, as e.g., Trimeniaceae, Himantandraceae, and Gomortegaceae (Table 1). Similarly, many of the families eliminated for limited accessibility have a woody habit (Table 1); these would have been eliminated for that character even if they had been more accessible (e.g., *Amborella trichopoda*). Plants with an aquatic habit, including water lilies (Nymphaeales: Nymphaceae, Cabombaceae) and *Ceratophyllum* (Ceratophyllales: Ceratophyllaceae), were eliminated due to the extensive cultivation requirements associated with maintaining large numbers of individual aquatic plants, as well as the difficulty in developing self-pollination and transformation protocols in an aquatic environment (Figure 2). Orders composed entirely of woody or aquatic plants were eliminated after review and consideration of the species comprising them.

The order Piperales (Aristolochiaceae, Hydnoraceae, Piperaceae, Saururaceae, Lactoridaceae) contains several herbaceous taxa (Table 1). Parasitic plants (Hydnoraceae) and those with highly reduced flowers (Saururaceae, Piperaceae) do not generally represent angiosperms, and so were eliminated (Figure 2). Hydnoraceae and Lactoridaceae would also be excluded due to limited availability (Table 1). Among the basal angiosperm families, only Aristolochiaceae contains highly accessible, easily cultivated herbaceous plants with features broadly representative of angiosperms in general.

### Aristolochiaceae candidates considered

**Figure 3:**
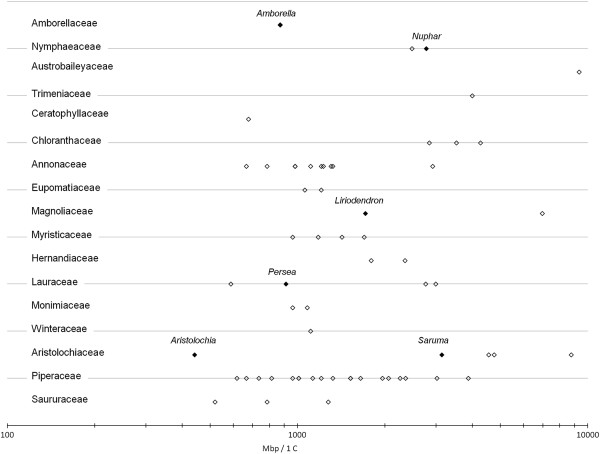
**Genome sizes in basal angiosperm families. **Bennett and Leitch [38] updated with Cui et al. [39], shown on logarithmic scale. Filled symbols indicate taxa used in The Floral Genome Project (http://www.floralgenome.org) or The Ancestral Angiosperm Genome Project (http://ancangio.uga.edu/), and for which EST resources are available. Compare basal angiosperm genome sizes to *Arabidopsis *at 125 Mb [40] and *Oryza* at 389 Mb [41]. The symbol representing *Aristolochia *is the proposed model *Aristolochia fimbriata*. Other species of *Aristolochia *have smaller genome sizes (see Figure 5, Table 4).

**Figure 4:**
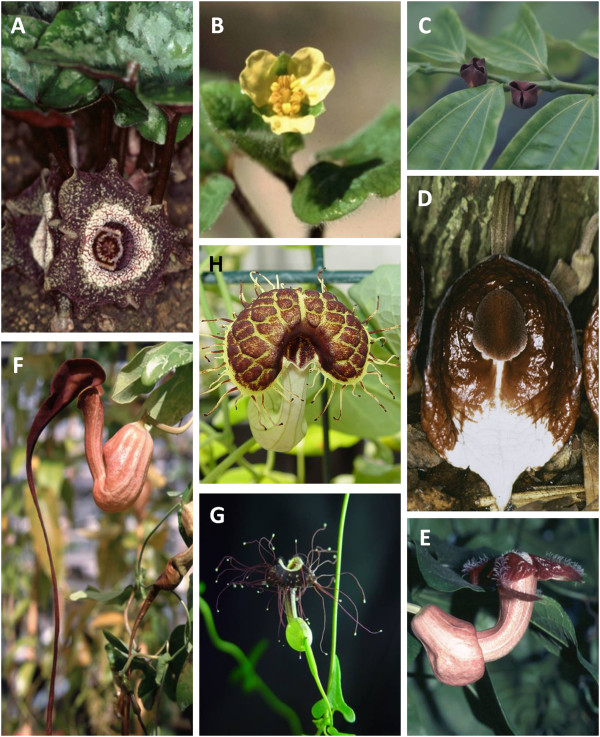
**Diversity of flower and growth forms in Aristolochiaceae. **Herbaceous perennials with radially symmetric 3-merous flowers include **A**. *Asarum chingchengense***B**. *Saruma henryi ***C**. *Thottea siliquosa*, a small shrub **D**. *A. arborea* (subgenus *Isotrema*), a tree-like shrub with flowers that mimic fungi **E**. *A. triactina *(subgenus *Pararistolochia*) **F**. *A. trilobata *(subgenus *Aristolochia*) with three lobed, evergreen leaves, grows as a vine with woody branches (liana) from which new growth emerges. **G**. *A. passiflorafolia *(subgenus *Aristolochia*) (photo used with permission from Changbin Chen) and H. *A. fimbriata *(subgenus *Aristolochia*).

We surveyed Aristolochiaceae, seeking species with rapid growth, no requirement for vernalization in the life cycle, ease of large scale cultivation, and a small genome size to facilitate gene function studies and genome sequencing and assembly (Figure 2). Members of genus *Aristolochia* have some of the smallest basal angiosperm genome sizes currently known (Figure 3). Therefore, we evaluated each genus in this family to identify the best species for model system development. Subfamily Asaroideae genera (*Asarum, Saruma*) (Figure 4A and B) require cold treatment to induce flowering, resulting in increased culturing efforts and extended time to flower, so they were eliminated (Figure 2). *Thottea,* here represented with *T. siliquosa* (Figure 4C)*,* was not possible to obtain for a detailed cultural survey (Table 2), as it can only be cultivated with very high maintenance, under a narrow range of conditions. Furthermore, it grows slowly, produces little tissue, few flowers, and few seeds. Of the *Aristolochia* species available for culturing, those requiring cold treatment (e.g. *A. serpentaria,* other members of subgenus *Isotrema* and *A. clematitis*, Figure 4D) were eliminated from consideration. Members of subgenus *Pararistolochia* (Figure 4E) were not available for culturing or are large vines with a long life cycle. Those species that did not bloom in six months (*A. californica, A. anguicida, A. macrophylla, A. tomentosa*), produced very few flowers (*A. trilobata, A. passiflorafolia*; Figure 4 F and G), or formed very large vines (*A. grandiflora*, *A. ringens*, *A. labiata, A. gigantea*) (Table 2, Figure 2) were also eliminated. The remaining candidates that met our criteria were two smaller members of subgenus *Aristolochia* (*A. elegans* and *A. fimbriata*), belonging to a group of subtropical and tropical species from South and Central America [18,37].

**Table 2:** Cultivation features for 25 Aristolochiaceae taxa considered

**Taxon**	**Floral productivity**	**Low maintenance**	**Dormancy required**	**Reported self fertile**^**1**^
ASAROIDEAE				
*Saruma henryi* Oliv.	+	++	facultative	
*Asarum canadense* L.	+	++	yes	
ARISTOLOCHIOIDEAE				
*Thottea siliquosa* (Lam.) Hou	+	+	no	
*Aristolochia* L				
*subgenus Isotrema*				
*A. serpentaria* L.	+	+++	facultative	
*A. macrophylla* Lam.	++	+	facultative	
*A. californica* Torr.	++		no	
*A. tomentosa* Sims	+++	+	facultative	
*A. holostylis* (Duchartre) F. Gonzalez	+	+	no	
*subgenus Pararistolochia*				
*A. goldieana* (Hook.f.) Hutch. & Dalz	+	++	no	
*A. prevenosa* F.Muell.	+	++	no	
*A. promissa* (Mast.) Keay	+	++	no	
*A. triactina* (Hook. f.) Hutch & Dalz	+	++	no	
*subgenus Aristolochia*				
*A. acuminata* Lam.	++	++	no	
*A. anguicida* Jacq.	++	++	no	
*A. clematitis* L.	+++	+++	yes	
*A. elegans* Mast.	++	++	no	++
*A. fimbriata* Cham.	+++	+++	facultative	++
*A. gigantea* Mart. & Zucc.	+	+	no	
*A. grandiflora* Sw.	+	+	no	
*A. passiflorafolia* Rich.	+	+	no	
*A. ringens* Vahl.	+	++	no	+
*A. trilobata* L.	+	++	no	
*A. lindneri* Berg.	+	+	no	
*A. maxima* Jacq	+	++	no	
*Aristolochia* sp.	+	++	no	

Observed presence of advantageous features indicated with plus sign. Observations were made either in the Biology Department greenhouse at Penn State University or at the Botanical Gardens of Dresden or Bonn.

^1^ after Petch [42].

### A phylogenetic perspective of genome sizes

**Figure 5:**
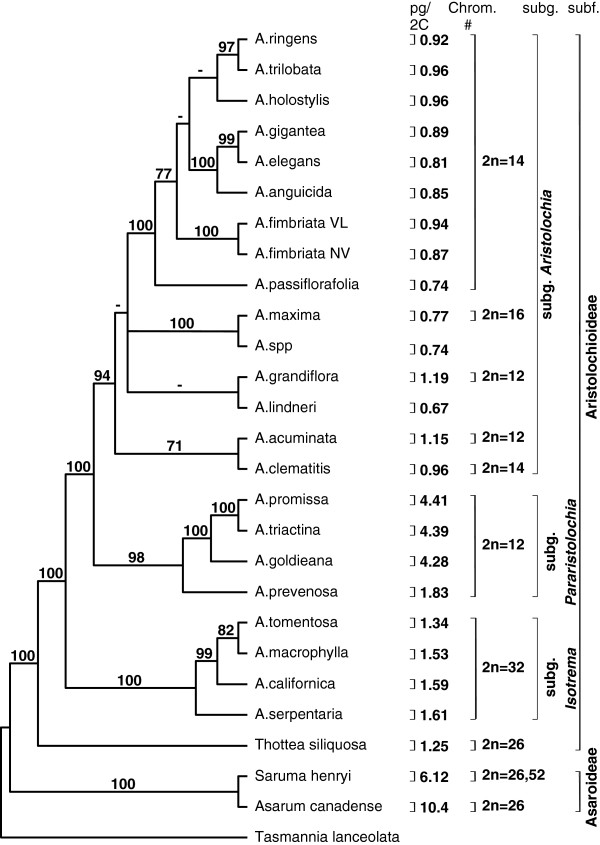
**Phylogenetic relationships among sampled Aristolochiaceae, with *Tasmannia lanceolata *(Canellales) as outgroup. **Maximum parsimony strict consensus tree with genome sizes (Mbp/1C) and chromosome counts indicated. If different genome sizes were obtained from different plants belonging to the same species, the smallest size was plotted on the tree. For range of genome sizes within one species and standard deviation refer to Table 4. Bootstrap values from 1000 replicates are indicated on the branches.

Because a small genome size facilitates molecular and functional investigation of genes of interest, including cloning and characterization of both coding and regulatory regions, and genome sequencing and assembly, our analysis of genome size evolution in Aristolochiaceae focused on species having small genomes, particularly in subgenus *Aristolochia*, for which we report here the smallest genome size to date from a basal angiosperm (*A. lindneri*) (Figure 5). In order to gain insight into the evolution of genome structure as well as genome size in Aristolochiaceae, chromosome numbers from previously published studies [43,44] were plotted along with genome sizes on a strict consensus tree using TreeGraph [45] (Figure 5, see also Additional file 1: Phylogram of Aristolochiaceae relationships).

A direct correlation between chromosome numbers and genome size for the family of Aristolochiaceae was not observed. Aristolochiaceae is subdivided into two subfamilies, Asaroideae and Aristolochioideae. Asaroideae, which has 2n= 26, 52 (*Saruma henryi*) and 2n=26 (*Asarum caudatum*) chromosomes, has about two to ten times the genome size of genus *Aristolochia* (Figure 5). *Thottea*, the earliest diverging branch in subfamily Aristolochioideae has the same number of chromosomes (2n=26) as *Asarum* and *Saruma*, but has only 1/9 and 1/5 of the genome size of *Asarum* and *Saruma,* respectively.

In contrast, within genus *Aristolochia*, species in subgenus *Aristolochia* exhibit the smallest genome sizes, but have a wide range of chromosome numbers. Species in subgenus *Isotrema* (Figure 4D) are generally characterized by 2n=32 chromosomes and have small genome sizes (554-774 Mbp mean genome size, 1C). Within subgenus *Pararistolochia* (Figure 4E) (1793-4321 Mbp mean genome size, 1C), which is sister group to subgenus *Aristolochia,* and therefore nested within the clade having species with small genomes, a lineage-specific increase in genome size can be seen. The increase is not associated with an increase in chromosome number, but in chromosome size [43]. The Australian species of *Pararistolochia* have less than half of the genome size of the African species, but still have the same number of chromosomes [43]. Within the subgenus *Aristolochia* (Figure 4F, G and H)*,* the different monophyletic groups recovered (Figure 5; see also Additional file 1: Phylogram of Aristolochiaceae relationships) are in accordance with previous studies [14,18]. It is interesting to note that *Asarum* and subgenus *Pararistolochia* have large chromosomes similar to those of monocots, whereas the remaining clades in Aristolochiaceae have small chromosomes [43]. *Aristolochia fimbriata* is a member of a clade of *Aristolochia* species with 2n=14 chromosomes and genomes roughly the size of *Oryza sativa*.

### Methods for genetics

We further evaluated selected taxa for self-compatibility and potential for genetic engineering, both of which are critical features of genetic systems. Self-pollination experiments were conducted with *A*. *elegans* and *A. fimbriata* because of their small genomes, ease of culture, and prolific flowering (Figure 2, Table 2). Both species could be hand pollinated to accomplish cross- and self-pollination events using simple methods, as described in Additional file 2: Cultivation Supplement. Morphological changes in the perianth and gynostemium associated with maturation of the anthers and stigmatic surfaces are described for *A. fimbriata* from the day of anthesis (day 1) through day 3 (Additional file 2: Figure S1). Self-pollination in *A. fimbriata* was most effective on day 2, both in terms of fruit production and seed viability (Additional file 2: Figure S2, Additional file 2: Table S3). Using *in vitro* germination methods, 59% of seeds produced in open-air pollinations of *A. fimbriata* germinated normally, compared to 50% of seeds produced from self-pollination of day 2 flowers (Additional file 2: Table S3). Further details are provided in Additional file 2: Cultivation Supplement.

**Figure 6:**
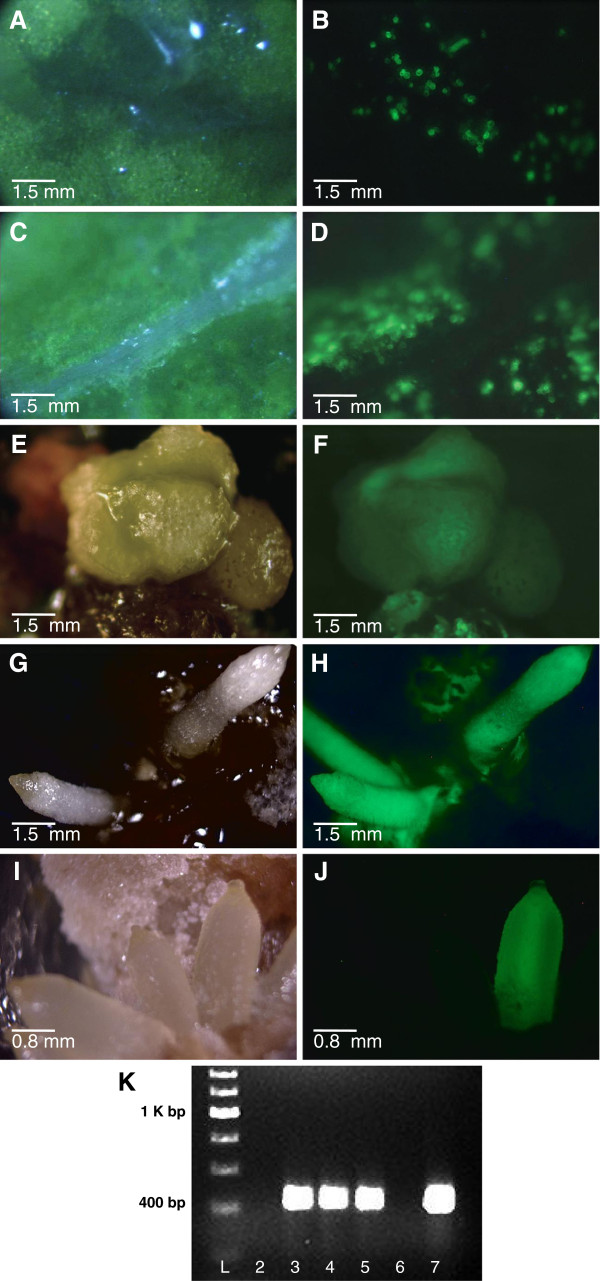
**Green fluorescent protein expression in Aristolochiaceae. A**, **C**, **E**, **G**, **I** . Light images. **B**, **D**, **F**, **H**, **J**. Fluorescent images. **A**-**D**. Leaf explants 10 days after *Agrobacterium tumefaciens *infection **A**, **B**. *Saruma henryi ***C**, **D**. *A. fimbriata ***E**, **F**. Regenerating *A. fimbriata* stem explant. **G**, **H**, **I**, **J**. Regenerating *A. fimbriata* roots (one root is shaded from light source in G). **K**. Gel image of PCR products; Lane L- 200bp ladder, bright band at 1K bp with corresponding bands at each 200bp; Lane 2- *A. fimbriata *(WT) DNA; Lane 3- *In vitro* transformed callus 1; Lane 4- *In vitro *transformed callus 2; Lane 5- *In vitro *transformed callus; Lane 6- Negative Control; Lane 7-Plasmid PC (1 ng/ul).

*Agrobacterium tumefaciens*-mediated genetic transformation experiments were performed with two varieties of *A. fimbriata* (NV, VL) and with *Saruma henryi*. The binary vector utilized [46] contained a neomycin phosphotransferase marker gene (NPTII) for antibiotic selection and an enhanced green fluorescent protein reporter gene (EGFP); both genes were under the control of the E12-Ω CaMV-35S promoter [46]. High frequency transient expression was observed in the leaf explants at 7 days after culture initiation (Figure 6A-D), followed by production of transgenic calli and shoot primordia at 24 days after culture initiation (Figure 6E and F). Stable transformation (evident 30 days after culture initiation) was observed in 40% of the *S. henryi* explants (n=25), and in 58% of the *A. fimbriata* explants (n=59). Additionally, transgenic calli, shoot, and root primordia (Figure 6G, 6H) were successfully regenerated from stem explants of *A. fimbriata* tissue cultured plants, with EGFP expression visible in a distinct subset of the regenerating tissues (Figure 6I and J). The integration of EGFP in the transgenic calli was confirmed by genomic PCR analysis (Figure 6K). The PCR reactions including DNA from green fluorescent calli (Figure 6K, lanes 3-5) resulted in the amplification of one 427 bp fragment identical to the control reaction including plasmid pGH00.0131 DNA (Figure 6K, lane 7). Amplification was not detected in the control reaction containing DNA from non-transgenic *Aristolochia* leaf tissue (Figure 6K, lane 2) or in the control reaction without DNA (Figure 6K, lane 6).

### Physical and life cycle features of Aristolochia fimbriata

**Figure 7:**
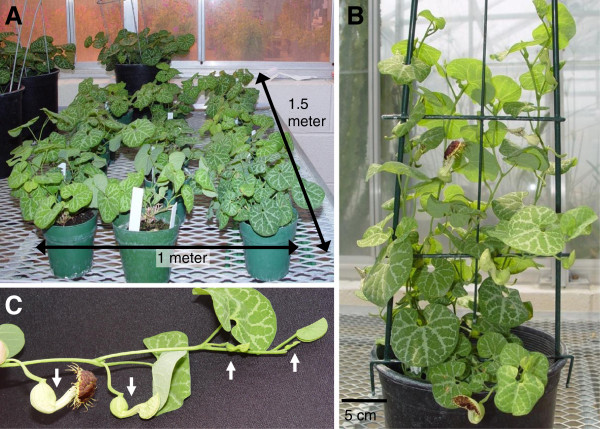
**Aristolochia fimbriata. A**. Twelve three-year old stock plants maintained in pots (12 cm diameter) occupy 1 m x 1.5 m bench space in the greenhouse **B**. Plants in use for genetic crosses, seed or tissue collection are trellised **C**. Close-up of vine showing flowers and floral buds at successive developmental stages on one stem [with arrows].

We characterized the physical and life cycle features of *Aristolochia fimbriata* (Figure 4H) to further assess its potential as a model system. Seeds planted in potting medium in the greenhouse germinated at rates up to 100%, and flowered in as few as 62 days after planting. Cultivated as a small pot crop in the greenhouse, *A. fimbriata* stock plants occupy minimal bench space (Figure 7A), and were not particularly susceptible to any pest or pathogen, though commercial pesticide treatments were applied greenhouse-wide as needed. Vines are supported by a small trellis during periods of flowering or fruit ripening (Figure 7B) to prevent mechanical damage and facilitate fruit harvest. Plants flower copiously from axillary nodes on multiple indeterminate stems that arise from the tuberous underground organ. A new flower opens every two to three days along the stem (Figure 7C) facilitating collection of staged tissues. Fruits from open-air pollinations on one-year-old plants averaged over one hundred seeds per capsule. Seeds are heart-shaped and small (~5x4x1 mm) and about 500 seeds can be stored in a 15-ml tube.

Although seasonally green in its native habitat, *A. fimbriata* grows year round in greenhouse culture. It forms a large perenniating tuber that can be divided to produce clones. Stems can be pruned back to the tuber after fruit collection to support greenhouse sanitation efforts, or “as needed” to stimulate new growth. New stems generated in this way will flower in two weeks. Fruit size, leaf size, and number of seeds increase in older, larger plants. The longevity of individual plants provides an ongoing source of seed from a single experimental subject.

We developed *in vitro* methods for germinating seeds to support collection of seedling tissues and for comparisons of seed viability. Light during germination is required for true leaves to emerge. Seeds germinated significantly better in wet toweling (mean= 61%) compared to germination on plates containing solid, sucrose-free media (mean=9%). Light and age of seed (up to 2.5 years) had no significant effect on germination with 80% germinating by 70 days. More details are available in Additional file 2: Cultivation Supplement.

### A large database of expressed gene sequences

The Ancestral Angiosperm Genome Project (http://ancangio.uga.edu/) has selected *Aristolochia fimbriata* for deep EST sequencing using a combination of traditional capillary [35] and extensive next generation (454, Illumina) sequencing of libraries constructed from multiple vegetative and reproductive tissues and stages. There are currently 36,248 Sanger EST sequences available from *A. fimbriata* libraries made from RNA from pre-meiotic flower buds (15,759 sequences) and a mixed library from multiple vegetative tissues (20,489 sequences). Over 3.8 million 454FLX ESTs are available from eleven non-normalized libraries representing pre- and post-meiotic floral buds, open flowers, early and late stage seedlings, terminal/axial buds, roots, young and mature leaves, and early and late stage developing fruits. Sequences have been cleaned, assembled, and posted to searchable public databases (see Methods).

Although detailed results using the *A. fimbriata* EST sequences will be presented elsewhere, we have initially characterized the Aristolochiaceae EST database here using BLAST analyses as described in Albert et al. [35] to identify putative homologs of many interesting regulatory and signaling genes (see Table 3 for examples). *Aristolochia* cDNA sequences had greater sequence similarity with other monocot or eudicot species (e.g., *Vitis, Poplar* and *Oryza*) than with *Arabidopsis*, highlighting the important role *A. fimbriata* will play in rooting phylogenetic analyses of gene function (Table 3, and additional results not shown). Consistent with the composition of tissues included in the libraries, we found putative orthologs of many genes important for development such as auxin efflux carrier PIN1, phytochrome signaling protein GIGANTEA, as well as floral development regulators AP3, AINTEGUMENTA, and SEP3 (Table 3). Maximum likelihood analyses of two genes reported in Table 3 illustrate contributions of *Aristolochia* to the interpretation of gene family evolution in angiosperms. Orthologs for alpha-galactosidase (Additional file 3: Maximum likelihood analysis of orthologs for alpha-galactosidase (ATAGAL1; AT5G08380)) identify an ancient gene duplication in a common ancestor of angiosperms (blue star). Orthologs for the MYB-domain protein AS1 in Arabidopsis, PHAN in *Antirrhinum*, and ROUGH-SHEATH in maize (Additional file 4: Maximum likelihood analysis of orthologs for MYB-domain protein ASYMMETRIC LEAVES 1 (AS1; AT2G37630)) identify an ancient gene duplication in a common angiosperm ancestor (blue star) and a second gene duplication specific to *A. fimbriata* (green star).

**Table 3:** **Orthologs of genes involved in development, cell wall biosynthesis, and stress response in *A. fimbriata *EST assemblies**

**Annotation**	**ESTs**	**Length**	**Identity**	**Evalue**	**AGI**
**Development**					
CLV2 protein kinase maintenance of stem cell populations (*Vitis vinifera*) Aristolochia|b3_lrc17313	69	1806	51%	5e-137	AT1G65380
GIGANTEA (*Vitis vinifera*) Aristolochia|b3_c14545	168	1770	58%	9e-157	AT1G22770
PIN1 auxin efflux carrier (*Arabidopsis thaliana*) Aristolochia|b3_lrc14465	123	1805	68%	0	AT1G73590
AINTEGUMENTA (*Carica papaya*) Aristolochia|b3_c20214	41	1991	84%	2e-161	AT4G37750
ASYMMETRIC LEAVES 1 (AS1), homologous to maize RS1 (*Vitis vinifera*) Aristolochia|b3_c2263	119	1553	50%	2e -93	AT2G37630
SEP3 MADs box transcription factor (*Vitis vinifera*) Aristolochia|b3_c2250	108	1131	70%	3e-105	AT1G24260
RNA Slicer that selectively recruits microRNAs and siRNAs (*Arabidopsis thaliana*) Aristolochia|b3_c16036	158	3157	82%	0	AT1G48410
NAC containing domain (*Vitis vinifera*) Aristolochia|b3_c613	521	1598	61%	2e-135	AT5G61430
AP3/APETALA 3 DNA binding/transcription (*Vitis vinifera*) Aristolochia|b3_c14320	154	1084	46%	6e -67	AT3G54340
**Cell wall biosynthesis**					
Cinnamoyl alchohol dehydrogenase, putative (*Vitis vinifera*) Aristolochia|b3_c675	405	1559	74%	8e-168	AT1G72680
ATAGAL1 alpha-galactosidase similar to ATAGAL2 (*Vitis vinifera*) Aristolochia|b3_c1141	231	2016	73%	2e-179	AT5G08380
Xyloglucan endotransglucosylase/hydrolase (XTH9) (*Populus trichocarpa*) Aristolochia|b3_lrc29524	21	783	77%	7e -45	AT4G03210
EXPA10: expansin involved in the formation of nematode-induced syncytia in roots (*Carica papaya*) Aristolochia|b3_c2232	340	1444	65%	3e-106	AT1G26770
**Stress response**					
BASIC CHITINASE in ethylene/jasmonic acid mediated signalling pathway during SAR (*Populus trichocarpa*) Aristolochia|b3_c857	301	1486	60%	1e -88	AT3G12500
Zeaxanthin epoxidase gene (*Arabidopsis thaliana*) Aristolochia|b3_c15151	113	1864	70%	0	AT5G67030
MAP KINASE 3 (MPK3) upregulated in response to touch, cold, salinity, chitin (*Carica papaya*) Aristolochia|b3_c14569	185	1655	78%	0	AT3G45640
POM-POM1; Chitinase-like protein essential for tolerance to heat, salt, drought stresses (*Vitis vinifera*) Aristolochia|b3_c843	723	1465	72%	1e-134	AT1G05850
Salt tolerance protein (STO) (*Populus trichocarpa*) Aristolochia|b3_c701	348	1661	52%	1e-171	AT1G06040
RAR1 disease resistance protein; Required for R protein accumulation (*Carica papaya*) Aristolochia|b3_c23	694	1245	64%	5e -93	AT5G51700

All assembled unigenes (http://ancangio.uga.edu/) were searched (blastx) against the sequenced plant genomes of *Arabidopsis thaliana, Oryza sativa, Populus trichocarpa, Vitis vinifera, Carica papaya, Medicago truncatula*, and *Sorghum bicolor* (http://www.floralgenome.org/tribedb/index.pl). Values reported are the number of ESTs comprising each unigene, the unigene length (bp), percent amino acid identity of the best blastx hit, e-value, Arabidopsis AGI, and the annotation of the best *Arabidopsis* hit as well as the species with the best overall hit following (in parentheses) and the *Aristolochia* unigene number.

## Discussion

### *Aristolochia fimbriata* has many characteristics of a valuable experimental model

**Figure 8:**
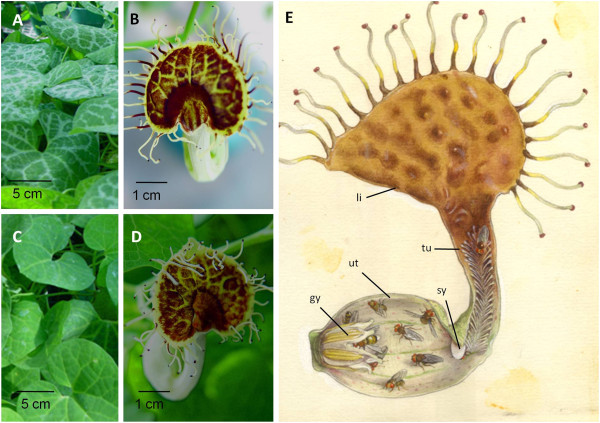
***Aristolochia fimbriata *genotype and perianth detail.A**, **B**. VL genotype **C**, **D**. NV genotype **A**, **C**. Presence, absence of leaf variegation **B**, **D**. Perianth varies in shape and color **E**. Perianth is highly modified for insect pollination. Modifications include limb (li), tube (tu), syrinx (sy), utricle (ut) and gynostemium (gy), which has stamen locules on the outside and interior stigmatic surfaces. Glass model by Leopold and Rudolph Blatschka made near Dresden, Germany illustrated by Fritz Kredel (reproduced with permission).

A basal angiosperm experimental system is needed to analyze basal angiosperm gene functions and to test hypotheses about the evolution of developmental and biochemical pathways in flowering plants. The herbaceous basal angiosperm *Saruma henryi* was selected for deep EST sequencing by the Floral Genome Project [35] and was initially considered for development as a model genetic system, but the slow growth, need for vernalization, and low flower and fruit production of *Saruma* prompted a thorough investigation of all other basal angiosperms, with the specific aim to identify a species more amenable to genetic experimentation. After evaluating 29 basal angiosperm families, including *in situ* assessment of over 20 species in family Aristolochiaceae and *in vitro* transformation of two species, we found in *Aristolochia fimbriata* many of the features desired in a genetic experimental system. *A. fimbriata* has the physical features for large-scale greenhouse cultivation, including robust container growth, continuous flowering, and self-compatibility, permitting the production of large numbers of homozygous individuals required for gene functional analysis in a single life cycle. We have begun to develop inbred lines to facilitate genome mapping and large scale mutagenesis experiments. High-efficiency transformation with a GFP construct allows rapid, nondestructive identification of transformed tissues for subsequent processing, and our *in vitro* micropropagation and regeneration methods can yield greenhouse acclimated plants in three months [47]. Individual *A. fimbriata* plants survive indefinitely, providing ongoing access to mutant lines that can be cloned for distribution to the research community. Currently available for analysis, the VL and NV genotypes possess a number of readily discernible traits including leaf variegation (Figure 8A and C) and perianth details (Figure 8B, D and E) amenable to investigation at the genetic level. Further inbreeding and crossbreeding of these and other genotypes can be used to dissect the genetic basis for phenotypic differences, yield useful markers, identify linked genes, and ultimately contribute to mapping and assembling the *A. fimbriata* genome sequence.

### *Aristolochia fimbriata* is well positioned for studies of the evolution of development

*Aristolochia fimbriata* is in a strong phylogenetic position to support comparative and evolutionary studies. Aristolochiaceae, with its approximately 550 species in four genera [18], is one of the most diverse and speciose families among the basal angiosperms. The largest genus in the family, *Aristolochia* contains approximately 450 species, and has long been of interest to botanists due to its monosymmetric, unipartite insect-trapping perianth (Figure 8E) and an unusual gynostemium, which is a structure formed by the fusion of the gynoecium with the anthers. *A. fimbriata* is a typical member of the family, such that its flower was modelled in glass by Leopold and Rudolf Blaschka in the latter half of the nineteenth century [48]. *A. fimbriata* differs from closely related *Aristolochia* species in several late stage modifications (e.g., fimbriae, papillae, pubescence), and in well-documented aspects of perianth and gynostemium development [49,50]. These features are potentially suitable for molecular analysis and studies of gene function using an *A. fimbriata* experimental system.

Comparisons between closely related taxa in Aristolochiaceae could support discovery of differences responsible for inter- and intra-generic speciation and may resolve long-standing questions about the origins of floral structures. Unlike the species-rich genus *Aristolochia*, the three other genera in the family (*Saruma* (Figure 4A), *Asarum* (Figure 4B), and *Thottea* (Figure 4 C)) have radially symmetric perianths, and account for approximately 100 species (Table 1). The bilaterally symmetric perianth of *Aristolochia* represents the most “basal” occurrence of this important floral adaptation in angiosperms. *A. fimbriata* presents the opportunity to investigate the genetic basis of bilateral symmetry in magnoliids and compare it with that found in the eudicot models *Antirrhinum*[51] and *Lotus*[52]. Existing microscopic studies of anatomy and development in Aristolochiaceae will facilitate comparative and functional studies, and provide insight into the evolution of development in this family. For *Saruma* the anatomy of stem, leaf, flower, and pollen have been described [53,54], and across Aristolochiaceae, ovule and seed development [55], microsporogenesis [56], female gametophyte evolution [15,57], and inflorescence morphology [58] have been described in detail.

Morphological and gene expression studies indicate Aristolochiaceae offers an excellent system in which to study the role of homologs of B-class MADS-box genes, which are required for the organ identities of petal and stamen in higher eudicots and putative homologous organs in grasses [28,59]. The flower of the monotypic genus *Saruma* (Figure 4B) with its apparent sepals, petals, stamens, and carpels resembles that of the typical magnoliid flower more than any other species in the family. Putative homologs of B-class genes (*AP3*, *PI*) are expressed in the stamens of *Saruma* and in its petaloid whorl which is of staminoid origin [60]. Putative *AP3* homologs have been found in *Thottea* and *Asarum*[61,62], and are expected to be expressed in the stamens and appendages opposite the sepals (which have been interpreted to be petaloid) in those genera [49,50,63-66]. In *Aristolochia,* putative B-gene homologs are expressed in the stamens, and also in the innermost, specialized cells of the outermost (and only) perianth whorl [67] which has been interpreted as a calyx [49,67]. Although the expression of putative homologs of the B-class genes in *Saruma* and *Aristolochia*[67] suggests they do not regulate perianth form by determining typical floral organ identity, expression is consistently found in pollinator-attracting structures of the flower. Homologs of B-class genes might regulate other aspects of perianth development and might not be required at all for perianth identity in Aristolochiaceae. Experimental evidence from Aristolochiaceae is needed to determine what role B-class homologs play in basal angiosperm floral development, particularly since the expression patterns of these genes in the first perianth whorl of other basal taxa is variable [61,68,69]. Gene function in Aristolochiaceae can be investigated in *A. fimbriata* using sequences from *Aristolochia* and three other genera in the family.

### *Aristolochia* contains highly developed biochemical pathways offering insight into evolution of biochemical synthesis and coevolution with insects

Aristolochiaceae produce a complex mixture of secondary metabolites, as is common in basal angiosperms. In particular, aristolochic acids and aristolactams are produced in Aristolochiaceae and are found throughout Piperales and the basal eudicots (reviewed in [70]). Compounds produced by alkaloid biosynthesis pathways in the poppy family (Papaveraceae, Ranunculales) are of great pharmacological importance, and it is a parallel pathway in Aristolochiaceae that yields aristolochic acids and aristolactams which are important for public health. Over 680 reports of traditional pharmacological use of about 100 species of Aristolochia have been compiled in a review [71]. The common name “birthwort” attributed to *Aristolochia* refers to traditional use of some species, particularly extracts from root tissues, as abortifacients, emmenagogues, or post-coital antifertility agents [72]. More recently, constituents of primarily root extracts from *Aristolochia* species have been isolated and evaluated for biological activity as antibiotics, antivenoms and tumor-inhibiting agents [73-80], although aristolochic acids become bioactivated and carcinogenic when ingested [81,82].

In addition to its pharmacological properties, *Aristolochia* provides an opportunity to explore coevolution of secondary metabolites with insects. Dipterans commonly serve as pollinators in *Aristolochia*[42,83-87], sometimes having specialized, mutualistic relationships involving egg deposition in the flowers [88-91]. Dipteran pollinators are thought to be attracted to secondary metabolites mimicking the aroma of a food source [42,91], or acting as pheromones to attract species-specific, sex-specific pollinators [92], or stimulating oviposition [89].

*Aristolochia* species are also important host plants for the larval stages of swallowtail butterflies (Papilionidae, Lepidoptera) [93-98]. Secondary metabolites found in particular *Aristolochia* species are critical for the defense and survival of associated swallowtail butterfly species during their feeding stage, such that the decline of butterfly populations is attributed to decreased distributions of particular *Aristolochia* species [99]. Finally, secondary metabolites of *Aristolochia* and related species are of interest for their repellent, insecticidal, and antifeedant activities in herbivorous plant pests [100-102]. Biochemistry in *Aristolochia* can be evaluated in greenhouse grown or micropropagated plants, using chemical analyses (e.g., mass spectroscopy, gas chromatography) to further characterize constituents of specific plant tissues in *A. fimbriata*. Biochemical pathways can be further investigated using transformed roots or callus (Figure 6G, H, I and J) currently available in the transformation-regeneration system, without further optimization.

### *Aristolochia* can provide insight into development of woodiness

Woodiness is an important seed plant feature, both for commercial and ecological purposes [9]. Growth forms within the Aristolochiaceae vary widely, presenting an opportunity to investigate growth form traits including flexibility, stiffness, and woodiness of closely related species [103]. Aristolochiaceae are most commonly perennial, self-supporting herbs (Figure 4A, B), procumbent or trailing, non-self-supporting vines (*A. passiflorafolia, A. fimbriata*) (Figure 4G, H), and woody lianas (Figure 4 F). Rarely, they are small woody shrubs (Figure 4C); and even more rarely trees (or tree-like forms) (Figure 4D). Early diverging members of the family (*Asarum* and *Saruma*) are characterized as perennial rhizomatous herbs, and the sister group to *Aristolochia* is comprised of the woody sub-shrub *Thottea* (Figure 4C). Perennial herbs appeared iteratively in the topology (Figure 5) of the family phylogeny and are nested within groups of woody vines. Most eudicots and monocots are modular organisms with indeterminate body plans. Shifts in the ontogenetic trajectory may be expected to have a profound effect on the overall size and potential life history of the descendant. This effect might have played a key role in growth form evolution and the development of flexibility, stiffness, and woodiness in Aristolochiaceae [103-105]. Differences in these growth form traits can be investigated in *Aristolochia* species of interest, including *A. fimbriata*, beginning with cellular level observations and descriptions of development of secondary growth (“wood”). Molecular and cell biological methods can be used to locate and describe in *Aristolochia* homologs of genes involved in growth form traits in other species (e.g., *Arabidopsis, Populus, Liriodendron*), to further characterize the role of interesting gene products in Aristolochiaceae.

### *Aristolochia* might reveal features of the ancestor common to monocots and eudicots

Aristolochiaceae occurs in Piperales, in the magnoliid clade, which is the most species-rich basal angiosperm clade and sister to the very large and diverse monocot plus eudicot clades [7-9]. As such, *Aristolochia* can provide a close outgroup for analysis of ancestral traits in the major groups of angiosperms. The ancient features of different lineages in Piperales have long been recognized, earning them classification as “paleoherbs” in early works [106,107]. In addition to being less woody than other basal angiosperm families, Aristolochiaceae and close relatives show a mixture of features of monocots and eudicots. Traits shared with eudicots include seedlings with two cotyledons and secondary growth from a vascular cambium. The more ancestral clade, Asaroideae, is comprised of rhizomatous perennials similar to basal monocots and eudicots. Also found in Piperales are aquatics (i.e.*,* Saururaceae), a common adaptation also found in both basal eudicots and monocots (e.g. Ranunculales, Acorales, Alismatales). Piperales, and Aristolochiaceae in particular, displays other features more commonly associated with monocots, including trimerous flowers, median prophylls (which are shared with nearly all monocots and only few eudicot clades), and subtype PII sieve-tube plastids [108,109]. Piperales also share distichous placement of leaves and palmate leaf venation with early diverging monocots (Alismatales, Arales). Indeed, in some phylogenies, Piperales appear as the closest relative to *Acorus*, the sister of all other monocots [110]. Consequently, many features in monocots and eudicots, both genetic and phenotypic, can be expected to have a homolog in Aristolochiaceae and its relatives, and would help to characterize the extinct ancestors of the eudicot, monocot and magnoliid clades.

### Growing genomic resources in Aristolochiaceae support further development of a model system

Genomic resources are growing rapidly for Aristolochiaceae, which will facilitate the identification and study of genes, gene families, and gene functions. Presently, over 3.8 million sequence reads from 13 diverse libraries are publicly available for *Aristolochia fimbriata* that provide a deep sampling of expressed gene sequences in this species. We anticipate these cDNA sequences, and others being currently generated, will contribute to new and ongoing studies of evolution of development in and comparative genomics with *Aristolochia*. Comparative analyses of cDNA sequences within Aristolochiaceae will be facilitated as well by 10,274 EST sequences from *Saruma henryi* (Figure 4B)*. Saruma* was selected by the Floral Genome Project (FGP) to represent Aristolochiaceae for floral transcriptome sequencing (http://www.floralgenome.org/taxa/) because it appears to display ancestral morphological characters in the family [53,111,112] and because its flower includes all four (sepal, petal, stamen, and carpel) floral whorls.

In addition to EST sequencing, two ongoing efforts have isolated micro RNAs (miRNAs) and other small RNAs from *A. fimbriata.* MiRNAs are small RNAs (21 nt) that play a major role as regulators of gene expression in various physiological, cellular, but, mainly developmental processes [113]. *A fimbriata* was selected as one of four basal angiosperms for small RNA sequencing using SBS/Illumina technology (http://smallrna.udel.edu/index.php). Our lab has used cloning and capillary sequencing as well as 454 pyrosequencing to sequence miRNAs ([114], unpublished data). This study allowed the isolation of hundreds of different miRNA sequences belonging to 32 conserved families as well as several non-conserved families. Several potential miRNA targets were found in cDNA sequences of *A. fimbriata*. Predicted target genes include transcription factors but also genes implicated in various metabolic processes and in stress defense. The isolation of miRNAs from *Aristolochia* presents a good opportunity for analyzing the function of miRNAs in basal angiosperms and for understanding how miRNA-mediated regulation of gene expression has evolved in land plants by comparing miRNAs in basal angiosperms to those in basal eudicots and lower land plants [115,116].

This core of genetic, genomic, and methodological resources is presently available as a foundation for further development of *Aristolochia fimbriata* as a basal angiosperm model system as well as for immediate use by the scientific community working on various areas of research including evolution of development, plant resistance to biotic and abiotic stresses, gene functional analysis, and comparative genomics.

## Conclusion

We have used a rigorous process to select and develop resources for the basal angiosperm, *A. fimbriata*. Culturing and hand pollination methods required for rapid generation of homozygous lines needed for genetic experiments are described. The small genome size and immediate availability of sequence data supports future studies of molecular genetics and evolution. Hypotheses about gene evolution and gene function can be tested using a reverse genetic approach, i.e., over and under expression studies in a transformable species suitable for large-scale cultivation. The transformation system we present supports experimental investigation of secondary metabolites, compounds for which *Aristolochia* and other basal angiosperms are well known and which have long been of interest for their pharmacologic properties and for their roles in the co-evolution of animals with plants. Optimizing the selection phase for transformed *A. fimbriata* explants would facilitate a high throughput transformation system for investigating gene function and evolution in a basal angiosperm. The development of virally-induced gene silencing (VIGS, [117]) would provide another valuable tool for functional analysis in *Aristolochia fimbriata*. Along with continued development of genetic tools and genomic resources, *A. fimbriata* has the potential to become an excellent experimental system to provide further insight into the developmental, structural, and biochemical diversity found among basal angiosperms.

## Methods

### Cultivation

We evaluated 24 species of Aristolochiaceae. These were selected to encompass the phenotypic plasticity of the four-whorled, actinomorphic, *Saruma*, the actinomorphic, single-whorled perianths of *Asarum* and *Thottea,* and the bilaterally symmetric, highly modified and diverse flowers of *Aristolochia.* The sampled taxa also reflected the genetic diversity of the whole family recovered by phylogenetic analysis. Plant material was obtained from commercial nurseries, private donations, and academic sources. Vouchers of specimens included in the phylogenetic analysis and sampled for genome sizes have been entered into herbaria as described in Table 4. For these species, we evaluated evolution of genome size and chromosome number in a phylogenetic context. For 14 species of *Aristolochia* we evaluated life cycle and cultivation characteristics. Plants were maintained in the Biology Department greenhouse at The Pennsylvania State University, University Park, PA. All seeds were germinated in soil-free potting medium (Pro-Mix BX, Premier Horticulture Inc., Quakertown, PA) in shallow germination trays with drainage holes, in the greenhouse at 18-27°C (varying from night to day) and 40-70% humidity. The trays were incubated on heating mats operating at approximately 27°C, as needed. Natural day length was supplemented with high-pressure sodium lamps (1000 watt) October through April to provide twelve-hour days. Plants received regular watering as needed. Depending on the stage of growth, regular fertilizer applications were provided, as a drench, alternating Peter's Professional 15-16-17 Peat Lite Special at 200 PPM nitrogen (once to twice weekly) with Peters Professional 21-7-7 Acid Special (Scotts Horticulture, Marysville, OH) at 200 PPM nitrogen (approximately every six weeks). The plants were drenched once a month with 100 ppm chelated iron (Sprint 330 10% iron, RoseCare.com, Santa Barbara, CA).

**Table 4:** Genome sizes, vouchers, sources, and accessions for sequence data used

**Taxon**	**Genome size (pg/2C) +/-std. dev. (n=4)**	**Std.**	**DNA content of sample species (Mbp/1C)**	**Voucher, herbarium**	**Source**	**GenBank Accession**
*Aristolochia acuminata *Lam.	1.15 +/-0.007	S	564	BJB06.06A, PAC	Victor Wong (private coll.)	
				Wanke & Neinhuis 146, DR	BG Dresden	DQ532063
*Aristolochia anguicida *Jacq.	0.81 +/-0.005	S	397	BJB06.03A, PAC	Mario Blanco (private coll.)	
	0.89 +/-0.011		436			
				Wanke & Neinhuis s.n., DR	BG Bonn	
*Aristolochia californica *Torr.	1.58 +/-0.006	S	774	BJB05.02A, PAC	Albert J. Hill (private coll.)	
	1.59 +/-0.011	R	779			
				Wanke & Neinhuis 143, DR	BG Dresden	DQ532039
*Aristolochia clematitis *L.	0.96 +/-0.001	S	470	BJB03.07A, PAC	Seneca Hill Perennials, NY	
	0.99 +/-0.001		485			
	0.90 +/-0.012	S	441	BJB03.03A, PAC	BG University Ulm	
	0.98 +/-0.008		480			
				W. Stahmüller, KL	Croatia, Is. Ilovik/Asinello	DQ296651
*Aristolochia elegans *Mast.	0.81 +/-0.007	S	397	BJB03.02A, PAC	Park Seed Company, cat. #0179-7	
	0.81 +/-0.004		397			
*Aristolochia fimbriata *Cham.	0.91 +/-0.004	S	446	BJB03.04A, PAC	Larry D. Rosen (“VL”) (private coll.)	
	0.97 +/-0.004		475			
	0.96 +/-0.001	S	470	BJB03.05A, PAC	Russ Strover (“VL”) (private coll.)	
	0.84 +/-0.009	S	412	BJB04.08A, PAC	Jardim Botanico, Departamento de Botanica, (“NV”), Universidade de Coimbra	
	0.89 +/-0.004		436			
*Aristolochia gigantea *Mart. & Zucc.	0.89 +/-0.013	S	436	BJB03.01A, PAC	Kartuz Greenhouses, CA	JX485569
	0.88 +/-0.009		431			
*Aristolochia grandiflora *Sw.	1.19 +/-0.009	S	583	BJB03.02A, PAC	Mario Blanco (private coll.)	
	1.20 +/-0.006		588			
	1.17 +/-0.008		573			
	1.21 +/-0.012		593			
	1.13 +/-0.012		554			
	1.26 +/-0.006		617			
	1.20 +/-0.006	R	588			
				Wanke & Neinhuis s.n., DR	BG Dresden	DQ532052
*Aristolochia goldieana *(Hook.f.) Hutch. & Dalz.	4.28 +/-0.113	S	2097	Neinhuis 117, DR	BG Dresden	
*Aristolochia holostylis *(Duchartre) F. Gonzalez	0.96 +/-0.029	S	470	Neinhuis 116, DR	BG Dresden	DQ532057
*Aristolochia lindneri *Berg.	0.67 +/-0.018	S	328	Neinhuis s.n., DR	BG Dresden, Bolivia, San Jose de Chiquitos	DQ532047
*Aristolochia macrophylla *Lam.	1.54 +/-0.011	S	755	BJB04.07A, PAC	Dawes Arboretum, OH	
	1.53 +/-0.033		750			
	1.52 +/-0.025	R	745			
	1.52 +/-0.018		745			
				Neinhuis s.n., DR	BG Dresden	DQ882193
*Aristolochia maxima *Jacq	0.77 +/-0.009	S	755	N.Pabon-Mora & F. Gonzalez, NY	NYBG	
	0.78 +/-0.017	R	764			
				Gonzalez 4018, COL	Panama, Panama	DQ532049
*Aristolochia. sp.*	0.74 +/-0.017	S	363	Wanke & Neinhuis s.n., DR	BG Munich	
*Aristolochia passiflorafolia *Rich.	0.74 +/-0.006	S	363	BJB06.05A, PAC	Mario Blanco (private coll.)	
				Neinhuis s.n., DR	Cuba, BG Dresden	
*Aristolochia prevenosa *F.Muell.	1.83 +/-0.006	S	1793	Neinhuis & Wanke s.n., DR	BG Dresden, Queensland, Australia, BG Dresden	
*Aristolochia promissa *(Mast.) Keay	4.41 +/-0.013	S	4321	Neinhuis 118, DR	BG Dresden	DQ532065
*Aristolochia ringens *Vahl.	0.93 +/-0.004	S	456	BJB06.07A, PAC	Mario Blanco (private coll.)	DQ532055
	0.91 +/-0.003		446			
*Aristolochia serpentaria *L.	1.69 +/-0.024	S	828	BJB03.03A, PAC	Larry D. Rosen (private coll.)	
	1.67 +/-0.019		818			
	1.57 +/-0.018	S	769	BJB05.01A, PAC	B&T World Seeds	
	1.56 +/-0.013		764			
	1.61 +/-0.035	R	789			
	1.58 +/-0.008		774			
				Priv. coll. B. Westlund	USA, Texas, Travis Co.	DQ532038
*Aristolochia tomentosa *Sims	1.13 +/-0.008	S	554	BJB03.06A, PAC	Seneca Hill Perennials, NY	
	1.39 +/-0.010		681			
	1.40 +/-0.022	S	686	BJB06.01A, PAC	Dawes Arboretum, OH	
	1.44 +/-0.011	R	706			
				Neinhuis 113, DR	BG Dresden	JX485570
*Aristolochia triactina *(Hook. f.) Hutch & Dalz.	4.39 +/-0.059	S	4302	Neinhuis 119, DR	BG Dresden	DQ532066
*Aristolochia trilobata* L.	0.91 +/-0.002	S	446	BJB04.04A, PAC	Kartuz Greenhouses, CA	
	0.99 +/-0.010		485			
	1.01 +/-0.002		495			
	1.02 +/-0.004	R	500			
*Asarum canadense* L.	10.19 +/-0.040	S	4993	BJB04.03A, PAC	Joel McNeal, (private coll.)	
	9.97 +/-0.083		4885			
	11.04 +/-0.307		5410			
*Saruma henryi* Oliv.	6.12 +/-0.044	T	2999	BJB06.08A, PAC	Heronswood Nursery, WA	
				Neinhuis 120, DR	BG Dresden	DQ532033
*Thottea siliquosa *(Lam.) Hou	1.25 +/-0.018	S	613	Neinhuis 121, DR	India, Kerala, BG Dresden	JN415679

Abbreviations: R, Rice (*Oryza sativa *ssp. *japonica *cv. ‘Nipponbare’ 0.9 pg/2C); S, Soybean (*Glycine max *cv. ‘Dunbar’ 2.35 pg/2C), T, Tobacco (*Nicotiana tabacum *cv.’SR-1’ 9.15 pg/2C); BG, Botanical Garden; PAC, Pennsylvania State University; DR, Dresden. Mbp DNA for plant species is based on the assumption 1pg=980 Mbp according to [118]. (BJB indicates first author).

### Genome sizing

Nuclear genome size estimations were obtained by flow cell cytometry following the protocol described by Arumuganathan and Earle [119]. The mean nuclear DNA content of each plant sample (expressed as pg) was based on 1000 scanned nuclei from sample tissue, compared to a preparation of tissue from the internal standard. Each nuclear preparation was sampled four times.

### Phylogenetic analysis

To clarify the phylogenetic positions of the taxa surveyed and to evaluate genome size in an evolutionary context we constructed a phylogenetic tree based on the plastid *trnK* intron and *matK* gene region. Total DNA was extracted using the CTAB method [120]. Vouchers, DNA, and tissue samples are stored at PAC. Amplification and sequencing was performed following methods described in detail by Wanke et al. [14] using published primers [14,18,121]. Sequences were manually aligned using PhyDE^®^[122]. Phylogenetic analysis was performed under maximum parsimony in PAUP* 4.0b10 [123] using PAUP scripts written by PRAP [124]. PRAP was used to implement the Parsimony Ratchet [125] following procedures described in Wanke et al. [14] but with 1000 ratchet replicates. Bootstrap values were additionally calculated to infer branch support with 1000 replicates. For an independent evaluation of relationships, a likelihood approach was chosen using the likelihood ratchet described by Morrison [126] as implemented in PRAP v. 2.0 [124] with default settings. A phylogram of the single maximum likelihood tree discovered with these methods indicates minimal branch length variation among the sampled *Aristolochia* species (Additional file 1: Phylogram of Aristolochiaceae relationships).

### Pollination experiments

Several species of *Aristolochia* had been reported to be self-compatible (*A. fimbriata*, *A. elegans*, *A. ridicula, A. ringens*) and generally protogynous [42], having a receptive stigma before the anthers dehisce. To determine if autogamous pollination could be successful, we attempted hand pollinations of *A. elegans* and *A. fimbriata* on day one (the day of anthesis), day two, day three, day four and day five. Unopened flower buds were covered with pollination bags prior to anthesis and observed daily. Hand pollination methods are detailed in Additional file 2: Cultivation Supplement. Pollinations were accomplished by severing the perianth midway across the utricle, just above the gynostemium (Additional file 2: Figure S2A). Pollen was transferred with a toothpick (Additional file 2: Figure S2B), and the remnant of the perianth was taped closed to prevent additional pollinator entry. Mature fruits (Additional file 2: Figure S2A) were collected and seeds germinated on wet toweling at 27°C exposed to 16/8 h day/night cycles.

### Genetic transformation

A protocol for genetic transformation of *A. fimbriata* was developed based on *in vitro* shoot regeneration [47] from leaf and stem explants coupled with *Agrobacterium*-mediated transformation. Leaf and internodal stem segments (2-3 cm long) from rooted tissue cultured *A. fimbriata* plants were excised and immediately immersed in induction media [46] to keep moist. The explants were inoculated with *Agrobacterium* strain AGL containing plasmid pGH00.0131 as previously described for *Theobroma cacao* L. [46]. After the inoculation the explants were blotted on sterile paper towels and co-cultivated on callus initiation medium (CI) [47] (0.5 mg/L 6BA, 1 mg/L NAA and 1mg/L TDZ) for 64 h in the dark at 27°C. Following co-cultivation, the explants were transferred to CI supplemented with 50 mg/L Geneticin (G418) (Cellgro, Herndon, VA) and 200 mg/L Claforan (Aventis, New Jersey) and incubated at 27°C in the dark for the remainder of the 14 days. After culture on CI medium, the explants were transferred to shoot regeneration medium (1.75 mg/L 6BA and 1.0 mg/L NAA) with 25 mg/L G418 and 200 mg/L Claforan and incubated in the dark for an additional 14 days. After the 28 days, the cultures were incubated under dim light until the development of shoot primordia. Individual glowing shoot primordia were then excised and transferred to REN2 medium (hormone free) in culture vessels (Sweetheart DSD8X and LDS58) under dim light conditions at 27°C [47] where they were maintained for further shoot elongation and rooting. EGFP fluorescence was observed and recorded as previously described [46]. Expression of red fluorescent protein in cells regenerating from transformed calli was independently observed in experiments by David Tricoli, U. C. Davis (personal communication).

### Genomic PCR analysis

The incorporation of the transgenes was confirmed by genomic PCR. A set of EGFP specific PCR primers were used for the analysis. The primers amplify a 427 bp EGFP fragment (5’-CCA GGA GCG CAC CAT CTT CT-3’ and 5’-CTC GTC CAT GCC GAG AGT GA-3’ [46]. Genomic DNA was isolated from non-transgenic *Aristolochia* leaf tissue and from transgenic calli from independent lines using a CTAB method [120]. Each PCR reaction (final volume of 20 μl) contained: 5 ng DNA (Qiagen purified DNA kit #69104), 10 μl JumpStart™ REDTaq^®^ ReadyMix (Sigma #0982), 5 μls water, forward and reverse primers at final concentration 0.5 μM. Reactions were prepared on ice. Control PCR reactions were also performed with 1 ng plasmid DNA from vector pGH00.0131 and PCR reaction mix without DNA. This represents an equal molar amount of plasmid DNA compared to the EGFP DNA contained in 5 ng total *Aristolochia* genomic DNA present in the leaf extract, assuming single copy/insertion of the EGFP gene. For the plasmid reactions, DNA was isolated using QIAGEN plasmid midi purification kit (QIAGEN Inc., Valencia, CA). PCR conditions for all reactions were: 94°C for 2 min, then 35 cycles of 94°C for 45 sec., 62°C for 45 sec, 72°C for 1 min. The final cycle was followed by incubation at 72°C for 7 min. 5 μl of each PCR reaction were loaded onto 1.5% high-resolution agarose gel (Sigma-Aldrich Co., St. Louis, MO, #A-4718) for electrophoresis.

### Expressed sequence EST datasets

To support evolutionary and functional studies in *Aristolochia* and related taxa, an extensive database of expressed gene sequences was produced for *Aristolochia fimbriata* by the Ancestral Angiosperm Genome Project (http://ancangio.uga.edu/). Seeds of second generation selfed individuals were germinated in a greenhouse to widely sample plant organs and developmental stages under standard growth conditions (see Additional file 2: Cultivation Supplement). RNAs were isolated using the RNAqueous^®^-Midi kit (Ambion, catalog #1911) following the manufacturer’s protocol (http://tools.invitrogen.com/content/sfs/manuals/cms_055263.pdf) with modifications as described in Carlson et al. [127]. Details of library preparation, RNA and cDNA quality control steps, and Sanger and 454flx sequencing will be described elsewhere.

Sequences from individual 454 libraries were extracted from SFF files and renamed to reflect the source material. The names of Sanger sequences also indicated the source library. After renaming, all sequences were combined into a single fasta file. All sequences in the combined fasta file were screened for contaminants and trimmed using seqclean (http://compbio.dfci.harvard.edu/tgi/software/) with the Roche library adaptors, the *Piper cenocladum* chloroplast genome (NCBI accession NC_008457), mitochondrial gene sequences from magnoliids *Calycanthus floridus*, *Liriodendron tulipifera*, *Laurus nobilis*, *Piper betle* and *Asarum sp.* Qiu 96018, and the Univec database (http://www.ncbi.nlm.nih.gov/VecScreen/UniVec.html). After screening and trimming, the 454 and Sanger sequences were assembled using MIRA version 3.0.5 (http://sourceforge.net/apps/mediawiki/mira-assembler) [128], with default settings for EST sequences. The resulting unpadded consensus sequences (i.e. unigenes) were compared to seven angiosperm proteomes using blastx. All sequences and assemblies are available at http://ancangio.uga.edu/content/aristolochia-fimbriata. Assemblies and blast results can be viewed through http://ancangio.uga.edu/ng-genediscovery/aristolochia.jnlp and the assembly can be searched using the Ancestral Angiosperm Genome Project blast interface at http://jlmwiki.plantbio.uga.edu/blast/blast.html.

## Competing interests

The authors declare they have no competing interests.

## Authors’ contributions

BB and SW contributed equally to the manuscript, specified criteria for a model system, provided herbarium material, tissue, and DNA vouchers, generated capillary sequence data and drafted the manuscript. BB developed hand pollination methods and inbred lines; specified tissue sampling for *A. fimbriata* libraries and developed transformation protocols. SW performed phylogenetic analyses. LL constructed *A. fimbriata* cDNA libraries for EST sequencing and contributed to transformation protocol development. LL and SW performed PCR verification of transformation. SWC generated EST sequence data, and SA, NW, AB, PKW, YJ, and JLM analyzed sequence data. YH performed tissue collection, RNA isolations, and contributed to transformation protocol development. PR performed tissue collections and RNA isolations and performed pollination and seed germination experiments. KA obtained cell flow cytometric estimates of genome sizes. CN contributed plant material and images and assisted in discussion of a basal angiosperm model plant. SNM contributed to transformation protocol development. HM participated in design and coordination and helped draft the manuscript. CWD conceived the study, and helped with experimental design and drafting the manuscript. All authors read and approved the final manuscript.

## Supplementary Material

Additional file 1 **Phylogram of Aristolochiaceae relationships. **Maximum likelihood analyses showing minimal variation in branch lengths within Aristolochiaceae. Only one maxium likelihood tree was found. Click here for file

Additional file 2 **Cultivation Supplement. **Description of perianth maturation, hand pollination methods, and self-compatibility in *A. fimbriata *and *A. elegans* includes: Figure S1 - *Aristolochia fimbriata *flower stages. Figure S2 - Hand pollination of *A. fimbriata. *Table S1 - Evaluating self-compatibility in *Aristolochia fimbriata. *Table S2 - Fruit set in *Aristolochia elegans* resulting from hand pollination. Click here for file

Additional file 3 **Maximum likelihood analysis of orthologs for alpha-galactosidase (ATAGAL1; AT5G08380). **Blue star indicates a gene duplication in a common ancestor of angiosperms. Click here for file

Additional file 4 **Maximum likelihood analysis of orthologs for MYB-domain protein ASYMMETRIC LEAVES 1 (AS1; AT2G37630). **Blue star indicates a gene duplication in a common ancestor of angiosperms, while the green star indicates a gene duplication in *A. fimbriata*. Click here for file
