# Supplementary material for: Characterization of the basal angiosperm Aristolochia fimbriata: a potential experimental system for genetic studies
Source: BMC Plant Biol. 2013 Jan 24;13:13. doi: 10.1186/1471-2229-13-13 (PMC3621149; doi:10.1186/1471-2229-13-13)
Supplement: Additional file 1 — Phylogram of Aristolochiaceae relationships. Maximum likelihood analyses showing minimal variation in branch lengths within Aristolochiaceae. Only one maxium likelihood tree was found. [file 1471-2229-13-13-S1.docx]

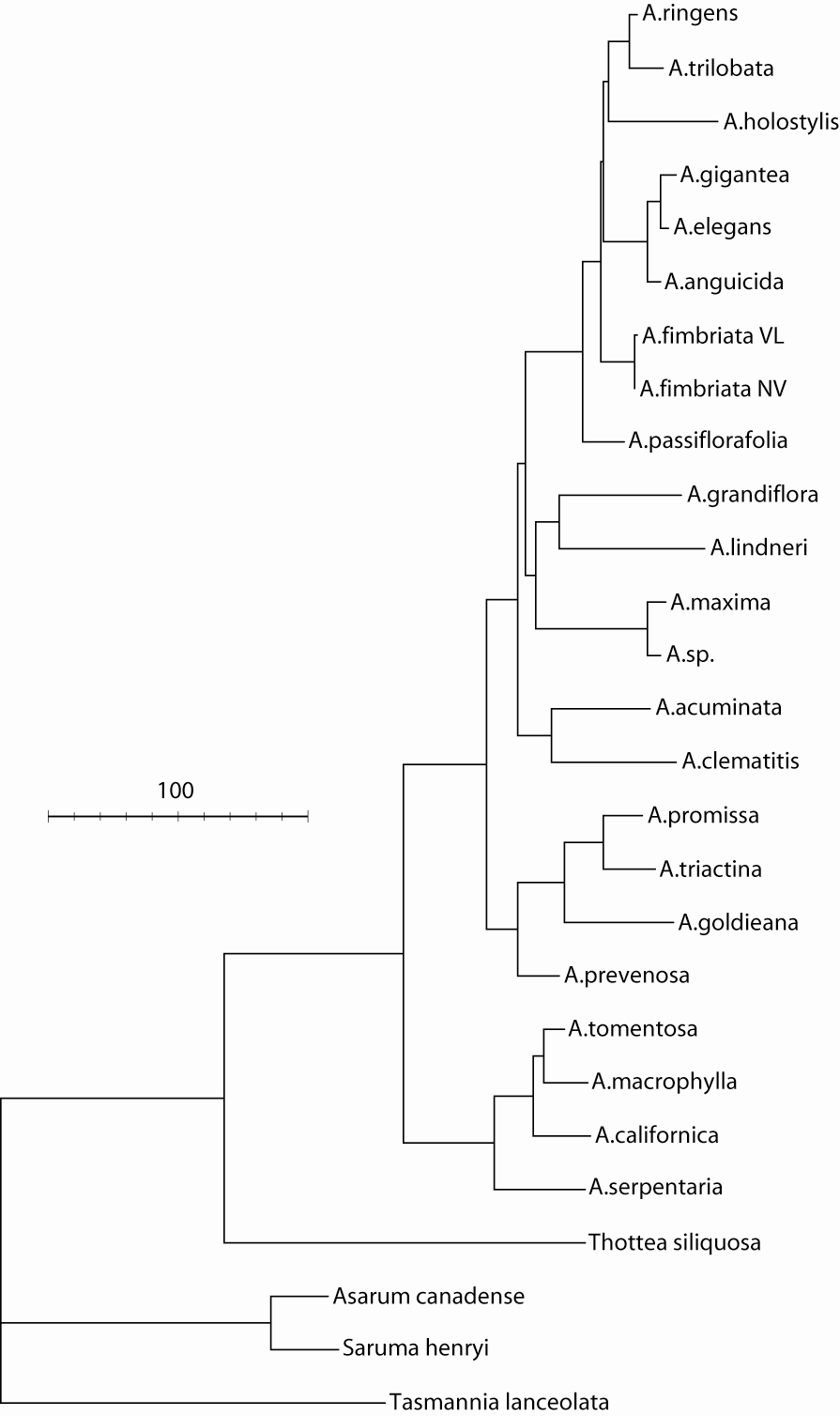


**Additional file 1 – Phylogram of Aristolochiaceae relationships.**

Maximum likelihood analyses showing minimal variation in branch lengths within Aristolochiaceae. Only one tree was found.
