# Supplementary material for: Characterization of the basal angiosperm Aristolochia fimbriata: a potential experimental system for genetic studies
Source: BMC Plant Biol. 2013 Jan 24;13:13. doi: 10.1186/1471-2229-13-13 (PMC3621149; doi:10.1186/1471-2229-13-13)
Supplement: Additional file 2 — Cultivation Supplement. Description of perianth maturation, hand pollination methods, and self-compatibility in A. fimbriata and A. elegans includes: Figure S1 - Aristolochia fimbriata flower stages. Figure S2 - Hand pollination of A. fimbriata. Table S1 - Evaluating self-compatibility in Aristolochia fimbriata. Table S2 - Fruit set in Aristolochia elegans resulting from hand pollination. [file 1471-2229-13-13-S2.docx]

# **Additional file 2 – Cultivation Supplement**

Description of perianth maturation, hand pollination methods, and self-compatibility in *A. fimbriata* and *A. elegans* includes:

**Figure S1 - *Aristolochia fimbriata* flower stages**

**Figure S2 - Hand pollination of *A. fimbriata***

**Table S1 - Evaluating self-compatibility in *Aristolochia fimbriata***

**Table S2 - Fruit set in *Aristolochia elegans* resulting from hand pollination**

# ***Background***

Several species of *Aristolochia* have been reported to be protogynous, and self-compatible (*A.* *fimbriata*, *A.* *elegans*, *A.* *ridicula, A. ringens* [1]; *A. bracteata* [2]; *A. inflata* [3]). Autonomous self-pollination has been reported in *A. paucinervis*, [4] and cleistogamy has been suspected in *A. serpentaria* [5]. Control of pollination events, including preventing unintended pollen delivery, is essential for reproducible genetic experiments. Therefore, we set out to 1) determine if pollination could be prevented, 2) document post-anthesis changes in the perianth and gynoecium useful for evaluating the receptiveness of the stigma and 3) identify the day on which hand pollinations would be most successful. We evaluated fruit production from autogamous pollination, in which both the pollen and stigmatic surfaces were from the same flower [6]. We also evaluated fruit production from geitogamous pollination, in which pollen was applied to stigmatic surfaces of a different flower on the same plant [6], selecting older flowers as pollen donor to better represent the presumed protogynous strategy used by *Aristolochia*. Results were compared with “outcrossings,” (xenogamous pollinations) [6] and fruit set on bagged flowers. We subsequently evaluated seed viability from fruits produced by self-pollination events, and compared that with the viability of bulk seed collected from open air pollinations. Open air pollinated fruit was collected from the general population of plants in greenhouse rooms housing both VL and NV cultivars along with a large community of pollinators, which were mostly common greenhouse “fungus gnats.” We confirmed reports of self-compatibility in *A.* *elegans* and *A. fimbriata,* and regularly germinated seeds up to three years old produced from self-pollinations and open-air pollinations.

# ***Perianth maturation***

The perianth of *A. fimbriata* opens early on the day of anthesis (Figure S1A), henceforth referred to as day one (D1). The lobes of the gynostemium of the D1 flower appear smooth, clean, and curved slightly inward (Figure S1D). The anthers, located on the outside of the gynostemium are closed, but a portion of an anther locule can be pried loose on D1 and placed onto stigmatic surfaces (located on the inside of the gynostemium) to effect self-pollination. The fimbriae of the limb of day two (D2) flowers are curved inward, covering the tube of the perianth d preventing the exit of any trapped pollinators (Figure S1B). The lobes of the gynostemium are no longer curved inward and pollen grains are visible dehiscing from the locules on the outer surfaces of the gynostemium lobes (Figure S1E). The loose pollen in D2 (and later) flowers can be collected on the tip of the toothpick. The perianth of the day three (D3) flower is beginning to visibly decline (Figure S1C). The D3 anther locules release pollen abundantly, the stigma has lost turgor, and the lobes of the gynostemium are beginning to recurve outward (Figure S1F).

**
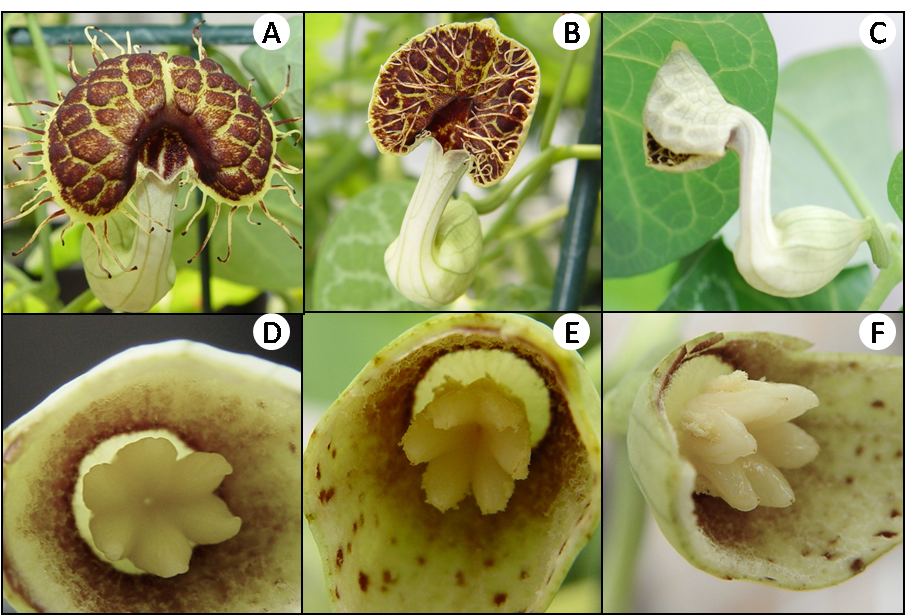
**

# Figure S1: Aristolochia fimbriata flower

**A.** Day one perianth at anthesis **B.** Day two perianth the day after anthesis **C.** Day three perianth **D.** Day one gynostemium; anthers have not dehisced **E.** Day two gynostemium; anthers have dehisced **F.** Day three gynostemium, pollen abundant.

# ***Hand pollination methods***

Selected flowers were enclosed in a pollination bag at an early stage of development to prevent pollinator access to the flower after anthesis. Each pollination bag was constructed of an approximately 12 cm x 30 cm piece of tulle (bridal veil) with an approx. 1.5 mm diameter pore size. The piece of tulle was folded in half, to create the closed end of the bag. The two long sides were sewn shut with a strong, durable, upholstery thread using 2 cm long stitches, leaving two drawstrings at the corners of the open, shorter side. Excess fabric was wrapped gently two to three times around a stem containing one or more flower buds, then the drawstring on the bag was tied around the stem below the petiole of the most proximal flower.


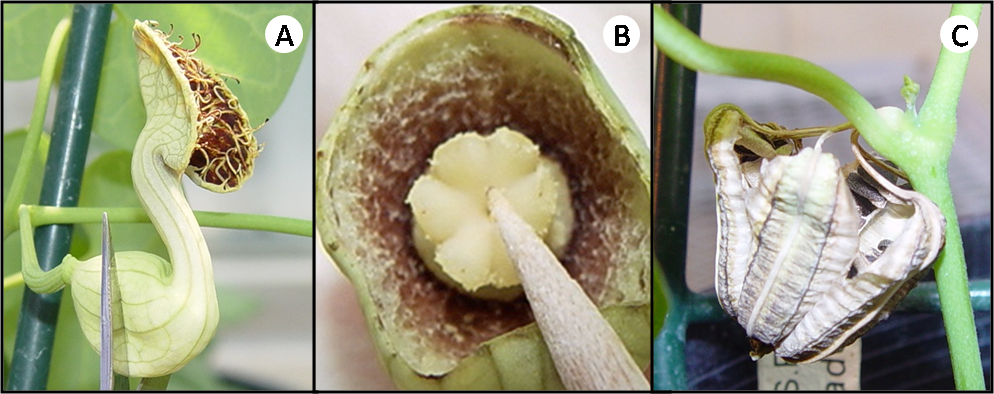


# **Figure S2: Hand pollination, A. fimbriata**

A. Sever the perianth midway through the utricle B. Apply pollen to stigmatic surfaces. (Seal to prevent further pollen delivery) C. Mature fruit dehiscing.

***Pollination is prevented with pollination bags***

Pollination bags prevented pollination in *A. fimbriata* except on one occasion, which represented less than one percent of all occurrences (Table S1). On that occasion, the perianth of the “not pollinated” flower producing fruit was lost, so it could not be inspected for the presence of a pollinator, which was likely responsible for the pollination event. Throughout the experiments, holes in the pollination bag and poorly tied bags were occasionally noted. Observation of an insect in the perianth at any time invalidated hand pollination efforts for that flower and it was not included in subsequent analyses.

# Table S1: Evaluating self-compatibility in A. fimbriata.

Day two autogamous pollination yielded greatest percent fruit set and germination success (bold).

| Self-pollination (autogamous) | Percent fruit set (sample size) ^1^ | Percent germination success (sample size) ^2^ |
| --- | --- | --- |
| D1 | 19.3 (57) | 28.0 (164) |
| D2 | **29.2 (154)** | **49.6 (733)** |
| D3 | 7.9 (76) | 29.9 (128) |
| D4 | 0 (12) | 0 |
| not pollinated | 0.8 (127)^3^ |  |
| not bagged |  | 59.0 (144) |

^1^Percent fruit set as a fraction of hand pollinations attempted, reported as sample size

^2^Percent germination success as a fraction of seeds collected, reported as sample size

# ***Day two autogamous pollinations are most successful***

Hand pollination in *A. fimbriata* was most successful on day two, both in terms of the numbers of seed pods produced from attempted pollinations and the viability of seed produced in those fruits (Table S1). Pods were produced from 29% of the hand pollinations attempted on day two, from 19% of the pollinations attempted on day one, and only 8% of hand pollination attempted on day three. The gynostemia of day four flowers were fragile and often broke, constraining efforts to attempt hand pollinations in flowers more than two days beyond the day of anthesis. Fruits produced by hand pollination contained a far more variable number of seeds than fruit produced by open air, insect pollinator-mediated pollination, which typically contained 10/15 seeds per carpel, six carpels per fruit. The reduced seed production in fruits from self (hand-) pollination events may be due to limited pollen transfer with hand pollination, or to mechanical damage.

***Self-compatibility in Aristolochia elegans***

Autogamous, geitonogamous, and xenogamous pollinations were evaluated using the hand pollination methods described for *A. fimbriata*, using very small sample sizes. Self pollinations of day one and day two flowers were very successful (Table S2). Viability of seed produced by hand-pollinations was not evaluated for *A. elegans.*

# Table S2: **Fruit set in *Aristolochia elegans* resulting from hand pollination**.

Percent fruit set (sample size) in *A. elegans* resulting from hand pollinations of flowers on days 1-4 with autogamous, geitonogamous and xenogamous pollen from flowers on days 1-3. Compare to 0% fruit set on 19 flowers, bagged before anthesis to prevent pollinator entry. Day 1 and 2 autogamous pollinations worked well in *A. elegans*, as did xenogamous pollinations (**bold**).

| Age of stigmatic surfaces **↓** | A g e o f p o l l e n s o u r c e | | | | | |
| --- | --- | --- | --- | --- | --- | --- |
|  | P o l l i n a t i o n t y p e | | | | | |
|  | Autogamous | | | Geitonogamous | | Xenogamous |
|  | D1 | D2 | D3 | D2 | D3 | D2 |
| D1 | **90 (10)** |  |  | 33 (3) |  | **67 (24)** |
| D2 |  | **80 (5)** |  |  |  |  |
| D3 |  |  | 20 (5) |  |  |  |
| D4 |  |  |  |  | 0 (1) |  |

***Seed germination methods***

We developed *in vitro* methods for germinating seeds to support collection of seedling tissues and for comparisons of seed viability. Seeds collected from fruits produced by open-air pollination of mixed VL and NV greenhouse stocks germinated significantly better in wet toweling packets in a growth chamber providing 16h days, 8h nights, 30°C days, 16°C nights than in sucrose-free media. Seeds incubated in 16h light/8 h dark did not germinate significantly better than seeds incubated in total darkness, but the first set of true leaves never developed in seedlings emerging in total darkness, thus the etiolated seedlings died (data not shown). However, seeds germinated most quickly in potting medium, with bottom heat, in the greenhouse (up to 95% in two weeks) than in wet toweling in a growth chamber. Furthermore, seedlings transferred from wet toweling to potting medium in the greenhouse after the first set of true leaves appeared typically bloomed two to three weeks later (at an older age) than did seeds germinated in potting medium in the greenhouse. This delay reflects the additional resources available to seeds germinating in potting medium, evident as a larger rooted seedling at four weeks than observed on seedlings germinated in toweling (data not shown).

***Effect of seed age on germination***

Using *in vitro* (in wet toweling, 25 seeds per replicate) germination, we evaluated the effect of age on seed viability and found no significant difference in total percent germination of seeds planted at seven day intervals, from 7 days after collection through 147 days after collection, and mean germination was 90% (n=15) by day 63 (data not shown). Furthermore, germination in 2.5 year old seeds was not significantly lower than in 2 year old or 1.5 year old seeds, and mean germination was 80% (n=12) by day 77.

***Additional literature cited***

1. Petch T: **Notes on *Aristolochia*.** *Annals of the Royal Botanic Gardens, Peradeniya* 1924, **VIII:**1-108.

2. Razzak MA, Ali T, Ali SI: **The pollination biology of *Aristolochia bracteolata* Lamk. (Aristolochiaceae).** *Pakistan Journal of Botany* 1992, **24:**79-87.

3. Sakai S: ***Aristolochia* spp. (Aristolochiaceae) pollinated by flies breeding on decomposing flowers in Panama.** *Am J Bot* 2002, **89:**527-534.

4. Berjano R, De Vega C, Arista M, Ortiz PL, Talavera S: **A multi-year study of factors affecting fruit production in *Aristolochia paucinervis* (Aristolochiaceae).** *Am J Bot* 2006, **93:**599-606.

5. Pfeifer HW: **Revision of the North and Central American Hexandrous Species of *Aristolochia* (Aristolochiaceae).** *Ann Mo Bot Gard* 1966, **53:**115-196.

6. Faegri K, Van Der Pijl L: *The Principles of Pollination Ecology.* 3rd edn. Oxford: Pergamon Press; 1979.
