# Supplementary figures and images for: Characterization of the basal angiosperm Aristolochia fimbriata: a potential experimental system for genetic studies
Source: BMC Plant Biol. 2013 Jan 24;13:13. doi: 10.1186/1471-2229-13-13 (PMC3621149; doi:10.1186/1471-2229-13-13)

Additional File 3A: AT5G08380

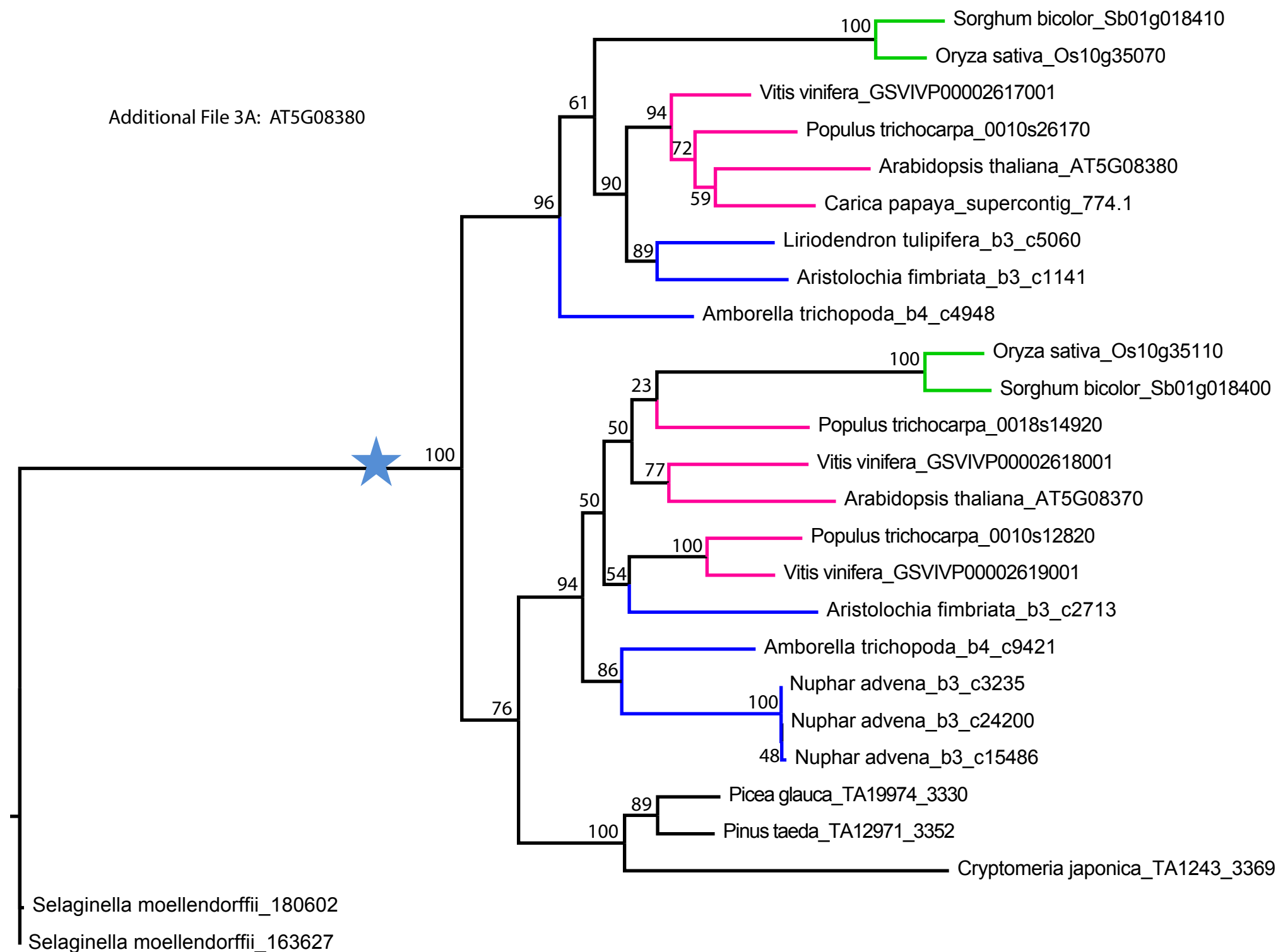

Supplement: Additional file 3 — Maximum likelihood analysis of orthologs for alpha-galactosidase (ATAGAL1; AT5G08380). Blue star indicates a gene duplication in a common ancestor of angiosperms. [file 1471-2229-13-13-S3.pdf]

Additional File 3B: AT2G37630

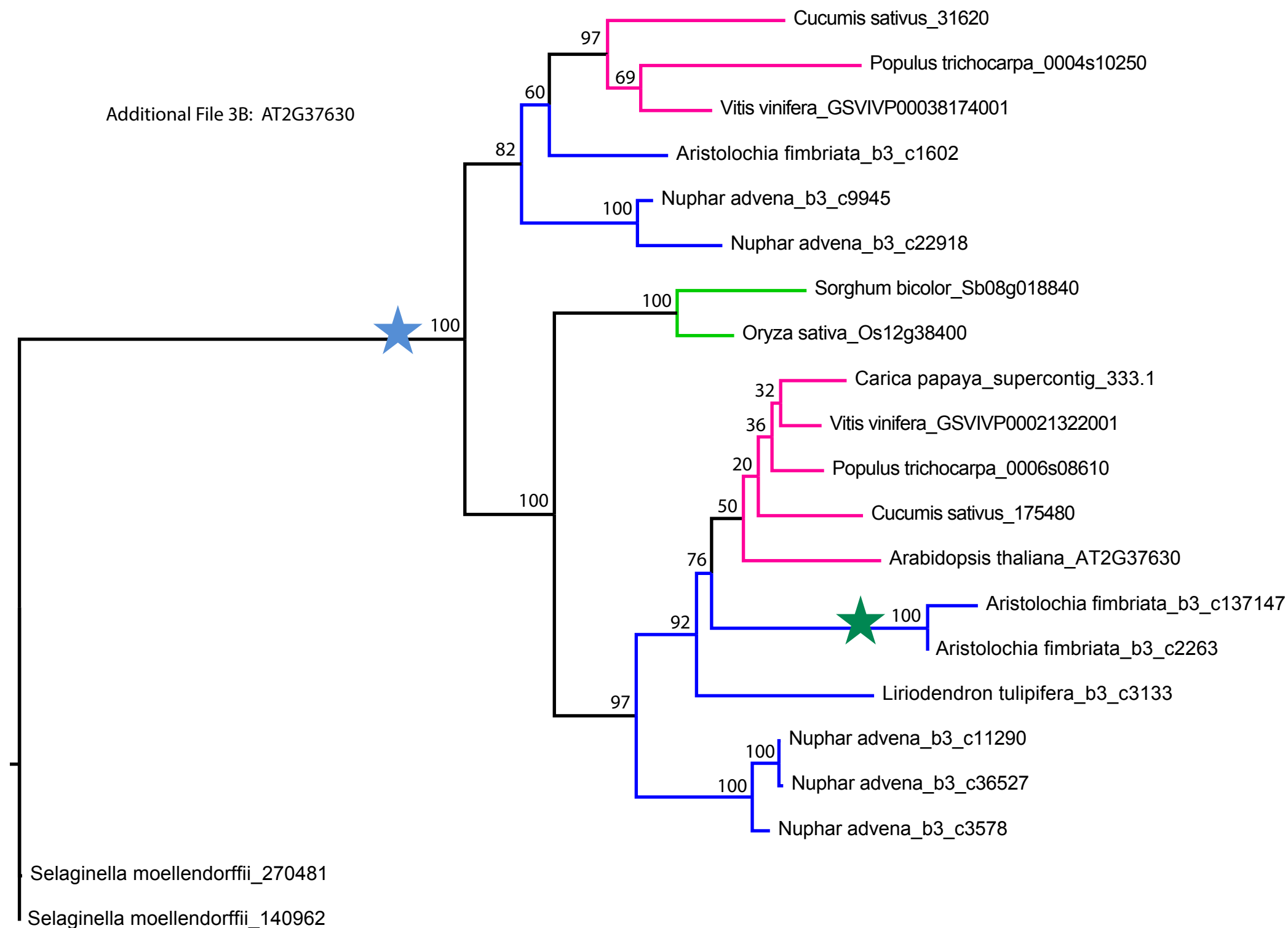

Supplement: Additional file 4 — Maximum likelihood analysis of orthologs for MYB-domain protein ASYMMETRIC LEAVES 1 (AS1; AT2G37630). Blue star indicates a gene duplication in a common ancestor of angiosperms, while the green star indicates a gene duplication in A. fimbriata. [file 1471-2229-13-13-S4.pdf]
